# Unconditional deep-water limit of the intermediate long wave equation in low-regularity

**DOI:** 10.1007/s00030-025-01037-7

**Published:** 2025-02-18

**Authors:** Justin Forlano, Guopeng Li, Tengfei Zhao

**Affiliations:** 1 School of Mathematics, The University of Edinburgh and The Maxwell Institute for the Mathematical Sciences, James Clerk Maxwell Building, The King’s Buildings, Peter Guthrie Tait Road, Edinburgh, EH9 3FD UK; 2 School of Mathematics and Statistics, Beijing Institute of Technology, Beijing, 100081 People’s Republic of China; 3 School of Mathematics and Physics, University of Science and Technology Beijing, Beijing, 100083 People’s Republic of China

**Keywords:** Intermediate long wave equation, Deep-water limit, Unconditional uniqueness, Well-posedness, Benjamin-Ono equation, 35Q53, 35A02, 76B55

## Abstract

In this paper, we establish the unconditional deep-water limit of the intermediate long wave equation (ILW) to the Benjamin-Ono equation (BO) in low-regularity Sobolev spaces on both the real line and the circle. Our main tool is new unconditional uniqueness results for ILW in $H^s$ when $s_0<s\le \frac{1}{4}$ on the line and $s_0<s< \frac{1}{2}$ on the circle, where $s_0 = 3-\sqrt{33/4}\approx 0.1277$. Here, we adapt the strategy of Moşincat-Pilod (Pure Appl Anal 5:285–322, 2023) for BO to the setting of ILW by viewing ILW as a perturbation of BO and making use of the smoothing property of the perturbation term.

## Introduction

We consider the Cauchy problem for the intermediate long wave equation (ILW):

1.1 $$\begin{aligned} \left\{ \begin{aligned}&\partial _tu- {\mathcal {G}}_\delta \partial _x^{2} u =\partial _x(u^2),\\&u|_{t=0}=u_0, \end{aligned} \right. \end{aligned}$$

where $0<\delta <\infty$, $u:{\mathbb {R}}\times {\mathcal {M}}\rightarrow {\mathbb {R}}$ and ${\mathcal {M}}={\mathbb {R}}$ or ${\mathbb {T}}= {\mathbb {R}}/(2\pi {\mathbb {Z}})$. Here, the operator ${\mathcal {G}}_\delta$ is defined by ${\mathcal {G}}_\delta ={\mathcal {T}}_\delta -\delta ^{-1} \partial _x^{-1}$, where ${\mathcal {T}}_\delta$ is given by

$$ \widehat{{\mathcal {T}}_{\delta } f}(\xi ) = -i \coth ( \delta \xi ) \widehat{f}(\xi ), \quad \xi \in \widehat{\mathcal {M}}, $$

$\coth (x)$ is the hyperbolic cotangent function defined by $\coth (x)=\frac{e^{x}+e^{-x}}{e^{x}-e^{-x}},$ $x\in {\mathbb {R}}\backslash \{0\},$ with the convention $\coth (x)-\frac{1}{x}=0$ for $x=0$, and $\widehat{\mathcal {M}}$ is the Pontryagin dual of ${\mathcal {M}}$, i.e., $\widehat{\mathcal {M}}={\mathbb {R}}$, when ${\mathcal {M}}={\mathbb {R}}$, and $\widehat{\mathcal {M}}={\mathbb {Z}}$, when ${\mathcal {M}}={\mathbb {T}}.$

ILW (1.1) was introduced in [20, 28] as a model for the propagation of the internal wave of an interface in a stratified fluid of finite depth $\delta$. It is completely integrable and is, in a sense, a physically relevant intermediary model between the Korteweg-de Vries equation (KdV) and the Benjamin-Ono equation (BO). Indeed, formally taking the shallow-water limit ($\delta \rightarrow 0$, and after a suitable rescaling), we have that (1.1) converges to KdV:

1.2 $$\begin{aligned} \partial _tv+ \partial _x^3 v =\partial _x(v^2). \end{aligned}$$

See Remark 1.6 for further details. In the deep-water limit ($\delta \rightarrow \infty$), we see directly that the operator ${\mathcal {T}}_{\delta }$ tends to the Hilbert transform ${\mathcal {H}}$ (with Fourier multiplier $-i\,sgn (\xi )$) and hence (1.1) reduces to the BO equation:

1.3 $$\begin{aligned} \partial _tv-{\mathcal {H}} \partial _x^2 v= \partial _x(v^2), \end{aligned}$$

see [1, 8, 29, 30, 39, 40].

The validity of such limits has been numerically observed [38] and their rigorous justification is one of the central important questions related to ILW; see [26, 40] for nice surveys. In this paper, we focus on the deep-water limit ($\delta \rightarrow \infty$) corresponding formally to the convergence of ILW to BO. Our main goal is to rigorously prove the unconditional convergence as $\delta \rightarrow \infty$ in low-regularity; see Theorem 1.1 and the discussion below.

Before we can hope to tackle this problem, we first need a satisfactory well-posedness theory for the limiting equation, namely BO, and the family of ILW equations for each $0<\delta <\infty$. Historically, this was a difficult problem as both ILW and BO are quasilinear [35] at every $L^2$-based Sobolev regularity, which nullifies any argument based on the contraction mapping theorem. Nonetheless, by now the well-posedness situation for BO on both ${\mathbb {R}}$ and ${\mathbb {T}}$ has been settled [6, 10, 11, 16, 17, 19, 22, 23, 27, 31–33, 36, 41]. Namely, BO is well-posed in $H^{s}({\mathcal {M}})$ for any $s>-\frac{1}{2}$ and this is sharp [4, 11]. In comparison, the situation for ILW is less well-understood, we refer to [1, 29, 34, 36]. Nonetheless, there have been significant breakthroughs in the low-regularity well-posedness of ILW. Ifrim-Saut [15] proved well-posedness in $L^2({\mathbb {R}})$ (and small data long-time dispersive decay estimates) by adapting the argument for BO in [16]. Namely, the authors in [15] use the BO gauge transform and the (quasilinear) normal form method. A key idea was to view ILW as a perturbation of BO in the following sense: after a Galilean transform which removes the term $\delta ^{-1}\partial _x$ (see (2.4)) we may write (1.1) as

1.4 $$\begin{aligned} \partial _tu-{\mathcal {H}} \partial _x^2 u= \partial _x(u^2)+{\mathcal {Q}}_{\delta } \partial _xu. \end{aligned}$$

The operator ${\mathcal {Q}}_{\delta } :=({\mathcal {T}}_{\delta }-{\mathcal {H}})\partial _x$ enjoys a strong smoothing property which suggests that this term is relatively harmless; see Lemma 2.3. Combining this idea with the unified $L^2$-well-posedness argument for BO from [33], the second author with A. Chapouto, T. Oh, and D. Pilod [8], established well-posedness in $L^2({\mathcal {M}})$ in both geometries ${\mathcal {M}}={\mathbb {T}}$ and ${\mathbb {R}}$.

Despite the absence of a scaling symmetry for ILW, the first and second authors, with A. Chapouto, T. Oh, and D. Pilod [7], identified $H^{-\frac{1}{2}}({\mathcal {M}})$ as the critical space for ILW with any depth parameter. They showed ill-posedness when $s<-\frac{1}{2}$ in the sense of failure of continuity of the data-to-solution map, and proved a-priori bounds on smooth solutions for $-\frac{1}{2}<s<0$. The latter result relied upon the complete integrability of BO, and interestingly not that of ILW. It remains an important open problem to establish the well-posedness of ILW in negative Sobolev spaces.

Let us now return to the question of the deep-water limit of solutions of ILW to those of BO as $\delta \rightarrow \infty$. Our main result in this regard is the following:

### Theorem 1.1

Let ${\mathcal {M}}={\mathbb {R}}$ or ${\mathbb {T}}$ and $s_0<s<\frac{1}{2}$, where $s_0:= 3-\sqrt{33/4}\approx 0.1277$. Given $u_0\in H^{s}({\mathcal {M}})$, let $\{u_{0,\delta }\}_{1\le \delta <\infty }\subset H^{s}({\mathcal {M}})$ such that $u_{0,\delta }$ converges to $u_0$ in $H^{s}({\mathcal {M}})$ as $\delta \rightarrow \infty$. Let $u\in ~C({\mathbb {R}};H^{s}({\mathcal {M}}))$ denote the unique global-in-time solution to BO (1.3) satisfying $u\vert _{t=0}=u_0$ and let $u_{\delta }\in C({\mathbb {R}};H^{s}({\mathcal {M}}))$ be the unique global-in-time solution to ILW (1.1) with depth parameter $\delta$ and $u_{\delta }\vert _{t=0}=u_{0,\delta }$, where the uniqueness for both the BO (1.3) and the ILW (1.1) solutions hold in the entire class $C({\mathbb {R}};H^{s}({\mathcal {M}}))$. Then, $u_{\delta }$ converges to *u* in $C({\mathbb {R}};H^{s}({\mathcal {M}}))$ as $\delta \rightarrow \infty$.^1^

Theorem 1.1 shows that the convergence in the deep-water limit $(\delta \rightarrow \infty )$ of ILW to BO occurs *unconditionally*: if $\{u_{\delta }(0)\}_{1\le \delta <\infty }$ converges to *u*(0), then the *entire* net of solutions to ILW $\{u_{\delta }\}_{1\le \delta <\infty }$, converges to *the* solution *u* of BO in the whole class $C({\mathbb {R}};H^{s}({\mathcal {M}}))$, irrespective of how $u_{\delta }$ or its limit *u* are constructed and of any additional auxiliary information on them. We note that the previous deep-water convergence results in $H^{s}({\mathcal {M}})$ for $s\ge \frac{1}{2}$, shown in [1, 8, 29, 30], do occur unconditionally since, as we discuss below, the unconditional uniqueness in $C({\mathbb {R}};H^{s}({\mathcal {M}}))$ of BO and ILW was known for any $s\ge \frac{1}{2}$.

In [8], the authors established that the deep-water limit occurs *conditionally* down to $L^2({\mathcal {M}})$: the ILW solutions they construct converge to those solutions of BO in [33]. We point out that when $s\le \frac{1}{4}$ on ${\mathcal {M}}={\mathbb {R}}$ or $s<\frac{1}{2}$ on ${\mathcal {M}}={\mathbb {T}}$, the convergence in Theorem 1.1 is not implied by their result since this convergence is predicated on the fact that their solutions also belong to some auxiliary function spaces and are not just members of $C({\mathbb {R}};H^{s}({\mathcal {M}}))$. We also refer to Remark 1.4 for a brief discussion on the regularity restriction in Theorem 1.1.

The key tool for upgrading the deep-water limit from a conditional to an unconditional statement is the unconditional uniqueness of solutions to ILW (1.1) (and BO) in $C({\mathbb {R}};H^{s}({\mathcal {M}}))$. Namely, given any $u_0\in H^{s}({\mathcal {M}})$, there is a unique solution to (1.1) in $C({\mathbb {R}};H^{s}({\mathcal {M}}))$ with initial data $u_0$, where uniqueness holds in the entire class $C({\mathbb {R}};H^{s}({\mathcal {M}}))$. The notion of unconditional uniqueness was introduced by Kato in [21] and implies that the solutions do not depend on how they are constructed. Here, solutions to (1.1) are understood to belong to $C([0,T]; H^{s}({\mathcal {M}}))$ and satisfy (1.1) in the sense of distributions on $(0,T)\times {\mathcal {M}}$ (and hence also (1.4)) for any $T>0$. In order for solutions to ILW to satisfy the equation (1.1) in the sense of distributions, we need $u(t)\in L^{2}_{\text {loc}}$, and thus we expect unconditional uniqueness for BO and ILW to hold down to $L^2$.

As BO is better understood compared to ILW, there are more unconditional uniqueness results for BO than for ILW. The well-posedness result of Burq-Planchon [6] gave unconditional uniqueness in $H^{\frac{1}{2}}({\mathbb {R}})$, followed by Molinet-Pilod [33] whose results lowered this to $s>\frac{1}{4}$ on ${\mathbb {R}}$ and $s\ge \frac{1}{2}$ on ${\mathbb {T}}$. On the circle, Kishimoto [25] pushed this down to $s>\frac{1}{6}$, which appeared to be almost sharp since the equation satisfied by the BO gauge transform has a cubic nonlinearity thus needing $u\in H^{\frac{1}{6}}\subset L^3$. Recently, Moşincat-Pilod [37] identified that this is not the case and established unconditional uniqueness in both geometries for $s> s_0$ where $s_0<\frac{1}{6}$. We will comment on their method of proof later.

For ILW, the general well-posedness result of Molinet-Vento [36] showed unconditional uniqueness for (1.1) for any $s>\frac{1}{2}$ on both the real line and the circle. As [8] adapts the approach in [33] to (1.4), they also obtained unconditional uniqueness in $H^{s}({\mathbb {R}})$ for $\frac{1}{4}<s\le \frac{1}{2}$ and $H^{\frac{1}{2}}({\mathbb {T}})$. In this paper, we further improve the unconditional uniqueness theory for (1.1) to the point that it agrees with that for BO on both ${\mathbb {R}}$ and ${\mathbb {T}}$.

### Theorem 1.2

Let ${\mathcal {M}}={\mathbb {R}}$ or ${\mathbb {T}}$, $0<\delta <\infty$, and $s>s_0$ where $s_0$ is as in Theorem 1.1. Then, ILW (1.1) is unconditionally globally well-posed in $C({\mathbb {R}};H^{s}({\mathcal {M}}))$. More precisely, for any initial data $u_0\in H^s ({\mathcal {M}})$, there exists a unique global-in-time solution *u* to the ILW (1.1) in the class $C({\mathbb {R}};H^s ({\mathcal {M}}))$ with $u\vert _{t=0}=u_0$.

Theorem 1.2 extends the known unconditional uniqueness on the line to $s_0<s\le \frac{1}{4}$ and to $s_0<s<\frac{1}{2}$ on the circle. Interestingly, these results show that $s=\frac{1}{6}$ is also not a barrier for the unconditional uniqueness of solutions to ILW.

With Theorem 1.2 in hand, we present a proof of Theorem 1.1. Let $u_{0}$ and $\{u_{0,\delta }\}_{1\le \delta <\infty }$ be as in the statement of the theorem. Let $u\in C({\mathbb {R}};H^{s}({\mathcal {M}}))$ be the solution to BO constructed in [17, 32, 33] with $u\vert _{t=0}=u_0$. By the unconditional uniqueness results in [37], *u* is unique solution to BO with this initial data in $C({\mathbb {R}};H^{s}({\mathcal {M}}))$. Similarly, let $u_{\delta }$ denote the solution to ILW such that $u_{\delta }\vert _{t=0} =u_{0,\delta }$ constructed in [8, 15]. This time, Theorem 1.2 guarantees that this is the unique solution with this initial data in $C({\mathbb {R}};H^{s}({\mathcal {M}}))$ for each fixed $0<\delta <\infty$. We then apply the conditional deep-water limit result in [8, Theorem 1.2] to obtain the convergence of $u_{\delta }$ to *u* in $C({\mathbb {R}};H^{s}({\mathcal {M}}))$.

We now briefly discuss the strategy of the proof of Theorem 1.2. We first consider solutions to (1.4), utilising the idea of viewing ILW as a perturbation of the BO equation. The general strategy is to then adapt the approach of Moşincat-Pilod [37] on unconditional uniqueness for BO (essentially, (1.4) with $\delta =\infty$), which itself is inspired by the argument of Kishimoto [25]. The goal is to compare two solutions *u* and $u^{\dag }$ to ILW with the same initial data.

First, we use (a very slightly modified form of) the gauge transform for BO introduced by Tao [41], and in the form by Burq-Planchon [6]. Namely, we pass from $u,u^{\dag }$ to their gauged variants $w, w^{\dag }$, respectively, defined in (2.8) (see also (2.4)), which satisfy (2.9) on ${\mathbb {R}}$. See Sect. 4 for the slightly different periodic setting. This equation is essentially the same as that for the gauged BO equation except for the addition of the new term $i\partial _x{\textbf{P}}_{+,hi } [ e^{iF} {\mathcal {Q}}_{\delta } v]$. It is not difficult to obtain control on the difference $u-u^{\dag }$ in terms of the difference $w-w^{\dag }$; see Lemma 2.8.

The main difficulty is obtaining the reverse: control on $w-w^{\dag }$ by $u-u^{\dag }$ with small constants; see Proposition 3.1. The proof of this result is responsible for the regularity restriction and is based on two normal form reductions (i.e. integration-by-parts in time), following those in [37]. We point out that the normal form method has been a powerful approach to showing unconditional uniqueness; see for example [2, 14, 24]. The key idea in [37] to go beyond the restriction $s>\frac{1}{6}$ is the addition of the refined Strichartz estimates which allows one to exploit the presence of a time integral for better integrability in space, see Lemma 2.6, and Lemma 4.1.

Whilst we follow the approach in [37], there are a few key differences. Firstly, we need to deal with the extra terms on the far right-hand side of (1.4) in view of the finite depth parameter $0<\delta <\infty$. This requires ${\mathcal {Q}}_{\delta }$ to be sufficiently smoothing; see Remark 1.5. Secondly, when ${\mathcal {M}}={\mathbb {R}}$, there are new issues, not present in [37], in justifying the normal form reductions. In the usual approach, one applies the identity $e^{i\phi t}=( i \phi )^{-1}\frac{d}{dt} e^{i\phi t}$, where we assume that $\phi =\phi (\xi ,\xi _1,\xi _2)$ does not vanish and $|\phi |$ is bounded from below, Fubini’s theorem, and integration-by-parts to show that

1.5 $$\begin{aligned} \begin{aligned}&\int _{0}^{t} \mathop {\mathrm {\int }}\limits _{\xi =\xi _1+\xi _2} e^{i \phi t'} f(t',\xi _1)g(t',\xi _2)d\xi _1 dt' \\  &=\mathop {\mathrm {\int }}\limits _{\xi =\xi _1+\xi _2} \frac{1}{i \phi } e^{i \phi t} f(t',\xi _1)g(t',\xi _2)d\xi _1 dt' \bigg \vert _{t'=0}^{t'=t} -\int _{0}^{t} \mathop {\mathrm {\int }}\limits _{\xi =\xi _1+\xi _2} \frac{1}{i\phi } e^{i \phi t'} \partial _t\big [f(t',\xi _1)g(t',\xi _2)\big ]d\xi _1 dt' \end{aligned} \end{aligned}$$

for $f,g\in C^{1}({\mathbb {R}}; L^2 ({\mathbb {R}}))$. Now, the $C^1$ assumption on both *f* and *g* allows us to use the product rule in time to distribute $\partial _t$ as

1.6 $$\begin{aligned} \partial _t\big [f(t,\xi _1)g(t,\xi _2)\big ] = (\partial _tf)(t,\xi _1)g(t,\xi _2) + f(t,\xi _1)(\partial _tg)(t,\xi _2). \end{aligned}$$

In our setting, we need to apply the above general computation with $f=\widehat{w}$, where *w* is the gauged solution in (2.8) with spatial Fourier transform $\widehat{w}$. However, the extra term $i\partial _x{\textbf{P}}_{+,hi } [e^{iF} {\mathcal {Q}}_{\delta } v]$ in (2.9) prevents us from showing $\widehat{w}(t,\xi )$ is continuously differentiable in time for each fixed frequency $\xi$; we refer to Remark 3.3 for further details. Consequently, we can no longer justify the product rule as in (1.6) as in the usual approach above. Inspired by [24, Appendix A], we proceed more carefully splitting the gauge variable into a good part (which is $C^1$-in-time) and a bad part (which is not). For the contribution due to the bad part, we prove the analogue of the identity (1.5) directly rather than through the integration-by-parts in-time followed by the product rule approach as above. As we do two rounds of the normal form reductions, we need this justification twice, first in Lemma 3.5 and secondly in Lemma 3.8.

As in Moşincat-Pilod [37], the arguments in this paper also imply a nonlinear smoothing estimates for the gauged variable *w*.

### Corollary 1.3

Let $s>s_0$ and $0<\delta <\infty .$ Let $u_0\in H^{s}({\mathcal {M}})$ and *u* be the unique solution to ILW (1.1) with $u\vert _{t=0}=u_0$. Let *w* denote the corresponding gauged variable defined by (2.8) and solving (2.9) when ${\mathcal {M}}={\mathbb {R}}$, or defined by (4.1) and solving (4.2) when ${\mathcal {M}}={\mathbb {T}}$. In the periodic setting, we further assume $\int _{{\mathbb {T}}} u_0 dx = 0$. Then, there exists $\varepsilon >0$ and $C:{\mathbb {R}}_{+}^{3}\rightarrow {\mathbb {R}}_{+}$ such that for any $T>0$, it holds that

$$\begin{aligned} \Vert w(t)- e^{-it\partial _x^2}w_0\Vert _{C([0,T]; H^{s+\varepsilon })}&\le C(T, \delta , \Vert u_0\Vert _{H^s}) \quad if \quad {\mathcal {M}}={\mathbb {R}}, \\ \Vert w(t)- e^{-it(\partial _x^2+m_0)}w_0\Vert _{C([0,T];H^{s+\varepsilon })}&\le C(T, \delta , \Vert u_0\Vert _{H^s}) \quad if \quad {\mathcal {M}}={\mathbb {T}}, \end{aligned}$$

where $$m_0 = \frac{1}{2\pi } \int _{{\mathbb {T}}} u_0^{2}(x)dx$$.

The proof of Corollary 1.3 follows from the smoothing estimates in Sect. 3, the resulting normal form equation for the gauged variable *w* in (2.8), and the uniqueness result of Theorem 1.2 which allows us to use the global-in-time a priori bound on the $H^s$-norm of solutions to ILW in [8, 15]. We refer the reader to the proof of [37, Corollary 1.2] for more details.

### Remark 1.4

As we can make sense of the nonlinearity in both (1.1) and (1.3) distributionally when the solutions belong to $L^2({\mathcal {M}})$ in space, we expect that the unconditional deep-water limit should also occur down to $L^2({\mathcal {M}})$. The regularity restriction $s>s_0$ in Theorem 1.1 is due to the restriction in Theorem 1.2. Thus, any improvement on the restriction of Theorem 1.2 yields an improvement in that of Theorem 1.1. In particular, it seems possible that further normal form reductions may lower the threshold $s_0$ closer to $s=0$.

### Remark 1.5

The approach of viewing ILW as a perturbation of BO is quite robust in the sense that we typically do not need to assume that the operator ${\mathcal {Q}}_{\delta }$ is infinitely smoothing. Indeed, in this paper, we only need smoothing of order $\frac{3}{2}+\varepsilon$, for any $\varepsilon >0$. Consequently, Theorem 1.2 and Theorem 1.1 extend to solutions of the ILW equation with two depth parameters, introduced in [28]:

$$\begin{aligned} \partial _tu- c_1{\mathcal {G}}_{\delta _1} \partial _x^{2} u-c_2{\mathcal {G}}_{\delta _2} \partial _x^{2} u =\partial _x(u^2), \end{aligned}$$

where $c_1,c_2>0$ and $0<\delta _1,\delta _2<\infty$. See also [7, Remark 1.3].

### Remark 1.6

The convergence in the shallow-water setting ($\delta \rightarrow 0$) requires a rescaling procedure, as introduced in [1]. Given a solution $u\in C({\mathbb {R}};L^2({\mathcal {M}}))$ to ILW (1.1), the rescaled function $\widetilde{u}(t,x) =\tfrac{1}{\delta } u(\tfrac{1}{\delta }t, x)$ solves the scaled ILW equation (sILW)

1.7 $$\begin{aligned} \partial _t\widetilde{u}-\tfrac{1}{\delta } {\mathcal {G}}_\delta \partial _x^{2} \widetilde{u} =\partial _x(\widetilde{u}^2). \end{aligned}$$

Note that *u* solves ILW (1.1) with initial data $\delta u_0$ if and only if $\widetilde{u}$ solves sILW (1.7) with initial data $u_0$. In particular, Theorem 1.2 implies that the sILW equation (1.7) is unconditionally globally well-posed in $C({\mathbb {R}};H^{s}({\mathcal {M}}))$ for $s>s_0$.

The shallow water limit to KdV is then taken with respect to the Cauchy problem for sILW, which has been rigorously proved in $H^{s}({\mathcal {M}})$ for $s>\frac{1}{2}$ [1, 29]. Moreover, as KdV (1.2) is unconditionally globally well-posed in $C({\mathbb {R}}; H^{s}({\mathcal {M}}))$ for any $s\ge 0$ and on both ${\mathcal {M}}={\mathbb {R}}$ and ${\mathbb {T}}$ [2, 42], the shallow-water limit for $s>\frac{1}{2}$ occurs unconditionally. Given any improvement in the regularity restriction for the shallow-water limit, our unconditional uniqueness result for sILW would immediately yield an improvement in the regularity restriction for the unconditional shallow-water limit.

Finally, we give an overview of the rest of this paper. In Sect. 2, we discuss the BO gauge transformation on ${\mathbb {R}}$ and how to adapt it to ILW (after a Galilean transform). We derive the gauged ILW equation and control certain harmless parts of its nonlinearity. We then discuss the refined Strichartz estimates adapted for ILW. In Sect. 3, we begin normal form reductions on the gauged equation, with the goal of proving Proposition 3.1 which controls the difference of gauged solutions. Lastly, in Sect. 4 we discuss the modifications to deal with the periodic setting.

## Gauge transform and Strichartz estimates

### Notation and preliminary estimates

We use $A\lesssim B$ to denote $A\le C B$ for some constant $C>0$. We denote the Fourier transform by ${\mathcal {F}}f(\xi ) = \widehat{f}(\xi ) = \int _{{\mathcal {M}}} e^{-ix\xi } f(x) dx$, for $\xi \in \widehat{{\mathcal {M}}}$, and endowed with the Lebesgue measure if ${\mathcal {M}}={\mathbb {R}}$ or counting measure if ${\mathcal {M}}={\mathbb {T}}$. For $s\in {\mathbb {R}}$, we denote the Sobolev space $H^s({\mathcal {M}})$ with the norm

$$ \Vert f\Vert _{H^s ({\mathcal {M}})} = \Vert J^s f\Vert _{L^2} =\Vert \langle \xi \rangle ^{s} \widehat{f}(\xi )\Vert _{L^{2}_{\xi }(\widehat{{\mathcal {M}}})}, $$

where $${\mathcal {F}}J^s f= \langle \xi \rangle ^s \widehat{f}(\xi )$$, with $$\langle \xi \rangle =(1+\xi ^2)^\frac{1}{2}.$$

Let $\psi :{\mathbb {R}}\rightarrow [0,1]$ be a smooth function with support in $[-2,2]$ and equal to 1 on $[-1,1]$. Define $\psi _N(\xi )=\psi (\frac{\xi }{N})-\psi (\frac{2\xi }{N})$. For $N\in 2^{{\mathbb {Z}}}$, we denote the Littlewood Paley projectors by

$$\begin{aligned} {\mathcal {F}}\,({\textbf{P}}_{\le N} f)(\xi ) = \psi (\tfrac{\xi }{N})\widehat{f}(\xi ), \quad {\mathcal {F}}\, {\textbf{P}}_N f = {\mathcal {F}}\,{\textbf{P}}_{\le N} f -{\mathcal {F}}\, {\textbf{P}}_{\le \frac{N}{2}} f =\psi _N \widehat{f}, \end{aligned}$$

and ${\textbf{P}}_{>N}:= 1-{\textbf{P}}_{\le N}$. We then set

2.1 $$\begin{aligned} \begin{aligned} {\mathcal {F}}\,( {\textbf{P}}_{+} f)(\xi ) = {\textbf{1}}_{\xi > 0} \widehat{f}(\xi ),&\quad {\mathcal {F}}\, ( {\textbf{P}}_{-} f)(\xi ) = {\textbf{1}}_{\xi < 0} \widehat{f}(\xi ),\\ {\textbf{P}}_{lo } = {\textbf{P}}_{\le 1}, \quad {\textbf{P}}_{LO }=P_{\le 2} \quad {\textbf{P}}_{hi }&=1-{\textbf{P}}_{lo } , \quad {\textbf{P}}_{+,hi } = {\textbf{P}}_{+} {\textbf{P}}_{hi } . \end{aligned} \end{aligned}$$

We also recall the frequency localised Sobolev embedding

$$\begin{aligned} \Vert {\textbf{P}}_N f\Vert _{L^{\infty } ({\mathcal {M}})} \lesssim N^{\frac{1}{2}}\Vert {\textbf{P}}_{N}f\Vert _{L^2 ({\mathcal {M}})}. \end{aligned}$$

#### Lemma 2.1

(Fractional Leibniz rule) Let ${\mathcal {M}}={\mathbb {R}}$ or ${\mathbb {T}}$, $s>0$ and $1<p_j,q_j,r\le \infty$, $j=1,2$, such that $\frac{1}{r}=\frac{1}{p_j}+\frac{1}{q_j}$. Then we have

$$\begin{aligned} \Vert J^s(fg)\Vert _{L^r ({\mathcal {M}})} \lesssim \Vert J^s f\Vert _{L^{p_1}({\mathcal {M}})} \Vert g\Vert _{L^{q_1}({\mathcal {M}})} + \Vert f\Vert _{L^{p_2}({\mathcal {M}})} \Vert J^s g\Vert _{L^{q_2}({\mathcal {M}})}. \end{aligned}$$

For a proof of Lemma 2.1, see [5, 9, 12] on ${\mathbb {R}}$ and [3, 13, 18] for ${\mathbb {T}}$. Moreover, we recall the following lemma for product estimates from [33, Lemma 2.7] and [8, Lemma 3.3].

#### Lemma 2.2

Let ${\mathcal {M}}={\mathbb {R}}$ or ${\mathbb {T}}$, $2\le q <\infty$, and $0\le s\le \frac{1}{2}$. Assume $F_1, F_2$ are two real-valued functions such that $\partial _xF_j =f_j$ for $j=1,2$, and $J^{s}g\in L^{q}$. Then

2.2 $$\begin{aligned} \Vert J^s (e^{iF_1} g)\Vert _{L^q }&\lesssim (1+\Vert f_1\Vert _{L^2}) \Vert J^s g\Vert _{L^q}, \end{aligned}$$

2.3 $$\begin{aligned} \Vert J^s \big ((e^{iF_1}-e^{iF_2}) g\big )\Vert _{L^q}&\lesssim \big ( \Vert f_1-f_2\Vert _{L^2} +\Vert e^{iF_1}-e^{iF_2}\Vert _{L^\infty }(1+\Vert f_1\Vert _{L^2}) \big ) \Vert J^s g\Vert _{L^q}. \end{aligned}$$

### Gauge transformation on ${\mathbb {R}}$

In this section, we slightly modify the gauge transformation for BO at low-regularity on the line from [6, 41] to deal with the ILW equation (1.1). The gauge transformation on ${\mathbb {T}}$ is simpler to construct due to the absence of very low frequencies and we discuss it later in Sect. 4.

Fix $0<\delta <\infty$. Let $s\ge 0$ and $u\in C([0,T]; H^s({\mathbb {R}}))=C_{T}H^{s}({\mathbb {R}})$ be a distributional solution to (1.1). In order to remove the transport term $\delta ^{-1}\partial _x$, we consider the Galilean transformation

2.4 $$\begin{aligned} v(t,x)=u(t,x+\delta ^{-1} t). \end{aligned}$$

Then, we have $v(t,x)\in C([0,T]; H^s ({\mathbb {R}}))$ and *v* is a distributional solution to

2.5 $$\begin{aligned} {\left\{ \begin{array}{ll} \partial _tv-{\mathcal {H}} \partial _x^2 v= \partial _x(v^2)+{\mathcal {Q}}_{\delta } \partial _xv, \\ v\vert _{t=0} =u_0, \end{array}\right. } \end{aligned}$$

where ${\mathcal {Q}}_{\delta } =({\mathcal {T}}_\delta - {\mathcal {H}})\partial _x$. We note here that ${\mathcal {Q}}_{\delta }$ is a smoothing operator. See [7, 8, 15].

#### Lemma 2.3

Let ${\mathcal {M}}= {\mathbb {R}}$ or ${\mathbb {T}}$ and $0<\delta <\infty$. For any $s\ge 0$, we have

$$\begin{aligned} \delta \Vert {\mathcal {Q}}_{\delta } f \Vert _{H^{s}({\mathcal {M}})}+ \delta ^{2} \Vert {\mathcal {Q}}_{\delta } \partial _xf \Vert _{H^{s}({\mathcal {M}})} \lesssim (1+\delta ^{-s}) \Vert f\Vert _{L^2 ({\mathcal {M}})}. \end{aligned}$$

To construct the gauge transformation following [6], we first construct a primitive of *v*. Namely, a function $F=F[v]$ such that $\partial _xF= v$. Let $\psi \in C_{0}^{\infty }({\mathbb {R}})$ and $\int _{{\mathbb {R}}} \psi (x) dx=1$. We set

2.6 $$\begin{aligned} F(t,x):=\int _{{\mathbb {R}}} \psi (y) \int _{y}^x v(t,z) dz dy+G(t), \end{aligned}$$

where

$$G(t):= \int _{0}^{t} \int _{{\mathbb {R}}} -\psi '(y) {\mathcal {H}}v(t',y) + \psi (y) {\mathcal {Q}}_{\delta } v(t',y) + \psi (y) v(t',y)^2 dy dt'. $$

It is clear that $\partial _xF=v$ and, moreover, from (2.5), *F* satisfies

2.7 $$\begin{aligned} \partial _tF- {\mathcal {H}} \partial _x^2 F=v^2+{\mathcal {Q}}_{\delta } v. \end{aligned}$$

Denote the Benjamin-Ono gauge transform of *F* by

2.8 $$\begin{aligned} W={\textbf{P}}_{+,hi } (e^{iF}) \quad \text {and} \quad w=\partial _xW= \partial _x{\textbf{P}}_{+,hi } (e^{iF})=-i {\textbf{P}}_{+,hi } (e^{iF} v). \end{aligned}$$

Then, as in [8, Section 3], we arrive at the gauged ILW equation:

2.9 $$\begin{aligned} \begin{aligned} \partial _tw+i \partial _x^2 w&= -2 \partial _x{{\textbf {P}}}_{+,hi } [(\partial _x^{-1} w)( {{\textbf {P}}}_-\partial _xv)] \\  &\quad -2\partial _x{{\textbf {P}}}_{+,hi } [({{\textbf {P}}}_{lo } e^{iF}) ( {{\textbf {P}}}_{-} \partial _xv)] + i\partial _x{{\textbf {P}}}_{+,hi } [e^{iF} {\mathcal {Q}}_{\delta } v] \end{aligned} \end{aligned}$$

with $w\vert _{t=0}= \partial _x{\textbf{P}}_{+,hi } [ e^{iF[u_0]}]$. The first two terms on the right-hand side of (2.9) are the usual terms appearing in the gauged BO equation, see [33, (2.11)]. The third term is the additional term arising from viewing the ILW equation as a perturbation of the BO equation. The smoothing property of ${\mathcal {Q}}_{\delta }$ in Lemma 2.3 ensures that this term is mostly harmless, like the second term on the right-hand side of (2.9). Indeed, we have the following.

#### Lemma 2.4

Let ${\mathcal {M}}={\mathbb {R}}$ or ${\mathbb {T}}$, $0<\delta <\infty$, $\sigma _1 \ge 0$, and $0\le \sigma _2< \frac{1}{2}$. Define

2.10 $$\begin{aligned} E_1(f,g)= -2\partial _x{\textbf{P}}_{+,hi } [({\textbf{P}}_{lo } f) ( {\textbf{P}}_{-} \partial _xg)] \,\, \text{ and } \,\, E_2(f,g)= i\partial _x{\textbf{P}}_{+,hi } [f {\mathcal {Q}}_{\delta } g]. \end{aligned}$$

Assume that $F_1,F_2$ are two real-valued functions such that $\partial _xF_j =f_j$, $j=1,2$. Then,

2.11 $$\begin{aligned}&\Vert E_1(e^{i F_1},g_1) -E_1(e^{iF_2},g_2) \Vert _{H^{\sigma _1}} \nonumber \\  &\lesssim [(1+\Vert f_1\Vert _{L^2})\Vert F_1-F_2\Vert _{L^{\infty }}+\Vert f_1-f_2\Vert _{L^2}] \Vert g_1\Vert _{L^2}+(1+\Vert f_2\Vert _{L^2})\Vert g_1-g_2\Vert _{L^2}, \end{aligned}$$

2.12 $$\begin{aligned}&\Vert E_2(e^{iF_1},g_1) -E_2(e^{iF_2},g_2) \Vert _{H^{\sigma _2}} \nonumber \\&\quad \lesssim \delta ^{-1}(1+\delta ^{-1-\sigma _2}) (1+\Vert f_1\Vert _{H^{\sigma _2}}+\Vert f_2\Vert _{H^{\sigma _2}})^2 \nonumber \\&\qquad \times \big [ \Vert g_1\Vert _{L^2}( \Vert f_1-f_2\Vert _{H^{\sigma _2}}+\Vert F_1-F_2\Vert _{L^{\infty }})+ \Vert g_1-g_2\Vert _{L^{2}} \big ]. \end{aligned}$$

Before we present the proof of Lemma 2.4, we recall the following from [37, Remark 2.3]. For a real-valued continuous function *F*, we always have $e^{iF}\in L^{\infty }({\mathbb {R}})\setminus L^{2}({\mathbb {R}})$. Thus, $e^{iF}$ is merely a tempered distribution and its Fourier transform is understood in the same sense. If $\partial _xF ~\in ~L^2({\mathbb {R}})$, then $\partial _x(e^{iF})\in L^2({\mathbb {R}})$ and so ${\textbf{P}}_{hi } (e^{iF})$ is well-defined through

2.13 $$\begin{aligned} {\textbf{P}}_{hi } (e^{iF}) = \partial _x^{-1} {\textbf{P}}_{hi } ( \partial _xe^{iF}). \end{aligned}$$

Note that (2.13) and Bernstein’s inequality then give

2.14 $$\begin{aligned} \Vert {\textbf{P}}_{hi } (e^{iF}) \Vert _{L^{\infty }_{x}} \lesssim \Vert \partial _xF\Vert _{L^{2}_{x}}. \end{aligned}$$

In general, ${\textbf{P}}_{lo } (e^{iF})$ may not be well-defined, so instead, with a slight abuse of notation, we define ${\textbf{P}}_{lo } (e^{iF}):= e^{iF} -{\textbf{P}}_{hi } (e^{iF})$. It then follows from (2.13) that

2.15 $$\begin{aligned} {\textbf{P}}_{lo } (e^{iF}) = 1+ {\textbf{P}}_{lo } (e^{iF}-1) + {\textbf{P}}_{hi } (e^{iF}-1) -{\textbf{P}}_{hi } (e^{iF}) = 1+ {\textbf{P}}_{lo } (e^{iF}-1), \end{aligned}$$

where $e^{iF}-1 \in L^2({\mathbb {R}})$ and so ${\textbf{P}}_{lo } (e^{iF}-1)$ is well-defined with the frequency projection ${\textbf{P}}_{lo }$ in (2.1). In particular, since ${\textbf{P}}_{+}{\textbf{P}}_{-}=0$,

2.16 $$\begin{aligned} E_{1}(e^{iF},g) = E_{1}(e^{iF}-1,g). \end{aligned}$$

#### Proof of Lemma 2.4

We first show (2.11). For $f\in L^2$, by frequency considerations, we have

2.17 $$\begin{aligned} E_{1}(f,g) = -2\partial _x{\textbf{P}}_{LO }{\textbf{P}}_{+,hi } [ ({\textbf{P}}_{lo } f)({\textbf{P}}_{LO }{\textbf{P}}_{-}\partial _xg)]. \end{aligned}$$

Then, by Bernstein’s inequality and (2.14), we have

$$\begin{aligned} \Vert E_1(e^{i F_1},g_1) -E_1(e^{iF_2},g_2) \Vert _{H^{\sigma _1}}&\lesssim \Vert {\textbf{P}}_{lo } (e^{iF_1}-e^{iF_2})\Vert _{L^{\infty }} \Vert g_1\Vert _{L^2} \\&\quad + \Vert {\textbf{P}}_{lo } (e^{iF_2})\Vert _{L^{\infty }} \Vert g_1-g_2\Vert _{L^2}. \end{aligned}$$

Now (2.11) follows from

$$\begin{aligned} \Vert {\textbf{P}}_{lo } (e^{iF_1}-e^{iF_2})\Vert _{L^{\infty }} \lesssim (1+\Vert \partial _xF_1\Vert _{L^2})\Vert F_1- F_2\Vert _{L^{\infty }}+\Vert \partial _xF_1-\partial _xF_2\Vert _{L^2}. \end{aligned}$$

We move onto proving (2.12). We will only prove the following inequality

2.18 $$\begin{aligned} \Vert E_{2}(e^{iF},g)\Vert _{H^{\sigma _2}} \lesssim \delta ^{-1}(1+\delta ^{-1-\sigma _2})(1+ \Vert f\Vert _{H^{\sigma _2}})^2 \Vert g\Vert _{L^{2}}. \end{aligned}$$

Then (2.12) follows from (2.18) and using similar ideas and (2.3). By (2.2), we have

$$\begin{aligned} \Vert E_{2}(e^{iF},g)\Vert _{H^{\sigma _2}}&\lesssim \Vert e^{iF} f {\mathcal {Q}}_{\delta } g\Vert _{H^{\sigma _2}}+\Vert e^{iF} {\mathcal {Q}}_{\delta } \partial _xg\Vert _{H^{\sigma _2}} \\&\lesssim (1+\Vert f\Vert _{L^2})\big (\Vert f {\mathcal {Q}}_{\delta } g\Vert _{H^{\sigma _2}}+\Vert {\mathcal {Q}}_{\delta } \partial _xg\Vert _{H^{\sigma _2}}\big ). \end{aligned}$$

For the second term, we use Lemma 2.3, while for the first term, by Lemma 2.1, Sobolev embedding, we have

$$\begin{aligned} \Vert f {\mathcal {Q}}_{\delta } g\Vert _{H^{\sigma _2}}&\lesssim \Vert f\Vert _{H^{\sigma _2}}\Vert {\mathcal {Q}}_{\delta } g\Vert _{L^{\infty }}+ \Vert f\Vert _{L^2} \Vert {\mathcal {Q}}_{\delta } J^{\sigma _2}g\Vert _{L^{\infty }}\lesssim \Vert f\Vert _{H^{\sigma _2}}\Vert {\mathcal {Q}}_{\delta } J^{\sigma _2+1}g\Vert _{L^2}. \end{aligned}$$

Another use of Lemma 2.3 completes the proof of (2.18).

#### Lemma 2.5

Let $0<\delta <\infty$ and $v,v^\dag \in C_TL^2 ({\mathbb {R}})$ be two solutions to (2.5) with initial data *v*(0) and $v^\dag (0)$, respectively. Assume that ${\textbf{P}}_{lo } v(0) ={\textbf{P}}_{lo } v^\dag (0).$ Let $F, F^\dag$ be the spatial primitives of $v, v^\dag$ constructed in (2.6) and satisfying (2.7), respectively. Then, we have

2.19 $$\begin{aligned} \Vert F(0)-F^\dag (0)\Vert _{L^\infty }&\lesssim \Vert v(0)-v^\dag (0)\Vert _{L^2}, \end{aligned}$$

2.20 $$\begin{aligned} \Vert F-F^\dag \Vert _{C_T L^\infty }&\lesssim \langle T \rangle (\Vert v\Vert _{C_TL^2} + \Vert v^\dag \Vert _{C_TL^2} +\delta ^{-1} ) \Vert v-v^\dag \Vert _{C_TL^2}. \end{aligned}$$

#### Proof

The proof is essentially the same as that in [33, Lemma 4.1], where the low-frequency assumption ensures (2.19) by Bernstein’s inequality. For (2.20), we use ${\textbf{P}}_{hi } ( F-F^{\dag })= \partial _x^{-1}{\textbf{P}}_{hi } (v-v^{\dag })$ and Bernstein’s inequality. For the low-frequencies, we use the Duhamel formulation (2.22). The only new term is that with ${\mathcal {Q}}_{\delta }$ for which we use Lemma 2.3, picking up a factor of $\delta ^{-1}$. $\square$

For $v\in C_{T}L^{2}$ which is a solution to

2.21 $$\begin{aligned} v(t)= e^{t {\mathcal {H}}\partial _x^2 } v(0) + \int _0^t e^{(t-t'){\mathcal {H}}\partial _x^2}&\big (\partial _x(v^2) +{\mathcal {Q}}_{\delta } \partial _xv \big ) dt' \end{aligned}$$

in the sense of spatial distributions for $t\in [0,T]$, we see that $F=F[v]$ in (2.6) solves

2.22 $$\begin{aligned} F(t)= e^{t {\mathcal {H}}\partial _x^2 } F(0) + \int _0^t e^{(t-t'){\mathcal {H}}\partial _x^2} \big (v^2+{\mathcal {Q}}_{\delta } v \big ) dt', \end{aligned}$$

in the sense of spatial distributions, for all $t\in [0,T]$, and where $F(0)= \int _{{\mathbb {R}}} \psi (y) \int _{y}^{x} v(0,z)dzdy$. However, the formal computations deriving (2.9) are only easily justified for sufficiently high-regularity $v\in C_{T}H^{s}$. For $v\in C_{T}L^2$, we need to justify that the gauged variable *w* is a distributional solution to (2.9). This can be done by following the arguments in [37, Remark 2.7], themselves originating in [25]. The idea is to consider frequency truncated $v_{\le N}:= {\textbf{P}}_{\le N} v$ for some $N\in 2^{{\mathbb {N}}}$, define a corresponding spatial antiderivative $F_{\le N}$ and gauged variable $w_{\le N}$, and to pass to the limit in $C_{T}H^{-2}_{x}$ as $N\rightarrow \infty$ in the resulting equation for $w_{\le N}$. In the presence of the depth parameter $0<\delta <\infty$, (2.12) with $\sigma _2=0$ allows to pass to the limit in $C_{T}L^2_{x}$ for the last term on the right-hand side of (2.9). Moreover, by Lemma 2.4, the gauge transformation *w* of the solution *v* solves

$$\begin{aligned} w(t)=e^{-it\partial _x^2 }w(0) + \int ^t_0 e^{-i(t-t')\partial _x^2 }&\big \{\hspace{-1mm} -\hspace{-1mm}2\partial _x{{\textbf {P}}}_{+,hi } [(\partial _x^{-1} w)( {{\textbf {P}}}_-\partial _xv)]\\  &\quad +E_{1}( e^{iF},v) + E_{2}(e^{iF},v)\big \} dt' \end{aligned}$$

in the sense of spatial distributions for each $t\in [0,T]$.

### Strichartz estimates

We recall the Strichartz estimates for BO on ${\mathbb {R}}$: if *u* solves $\partial _tu-{\mathcal {H}}\partial _x^2 u=f,$ on $[0,T]\times {\mathbb {R}}$, then, for any pair (*p*, *q*) satisfying $4\le p\le \infty$, $2\le q\le \infty$, and $\frac{2}{p}+\frac{1}{q}=\frac{1}{2}$, we have

$$ \Vert u\Vert _{L^p_T L_{x}^q } \lesssim \Vert u\Vert _{L^\infty _T L_{x}^2} +\Vert f\Vert _{L^1_TL_{x}^2}. $$

We call such pairs of exponents (*p*, *q*) Strichartz admissible. We now state the refined Strichartz estimates we will use here, which are essentially proven in [37, Lemma 2.8] with the addition of using Lemma 2.3.

#### Lemma 2.6

(Refined Strichartz estimates) Let $0<\delta <\infty$, $0\le s \le \frac{1}{4}$, $N\in 2^{{\mathbb {N}}}$, $N\ge 2^6$, and $T>0$. Let (*p*, *q*) be a Strichartz admissible pair and define $\alpha (s,p)=\frac{1}{p}(\frac{3}{2}-s)-s$.

(i) If *v* is a solution to the equation $\partial _tv-{\mathcal {H}}\partial _x^2 v = \partial _x(v_1v_2+v_3v_4) + {\mathcal {Q}}_{\delta } \partial _xv_5$, then

$$\begin{aligned} \begin{aligned} \Vert {\textbf{P}}_N v \Vert _{L^p_T L^q}&\lesssim T^{\frac{1}{p}} N^{\alpha (s,p)}\big \{ \Vert {\textbf{P}}_N v\Vert _{L^\infty _T H^s } + \Vert v_1\Vert _{L^\infty _T H^s } \Vert v_2\Vert _{L^\infty _T H^s } \\&\quad + \Vert v_3\Vert _{L^\infty _T H_x^s } \Vert v_4\Vert _{L^\infty _T H^s }+ \delta ^{-2}\Vert {\textbf{P}}_N v_5\Vert _{L^\infty _T L^2} \big \}. \end{aligned} \end{aligned}$$

(ii) If *w* is a solution to the equation

$$\partial _tw+i \partial _x^2 w = -2 {\textbf{P}}_{+,hi } \partial _x[ \partial _x^{-1} w_1 \cdot {\textbf{P}}_- \partial _xw_2 +\partial _x^{-1} w_3\cdot {\textbf{P}}_- \partial _xw_4 ] + \phi , $$

where $\mathop {\textrm{supp}}\limits \widehat{w}_1, \mathop {\textrm{supp}}\limits \widehat{w}_3 \subset (2^{-3},\infty )$ and $\phi \in L^{\infty }_{T}L^{2}_x$, then we have

2.23 $$\begin{aligned} \begin{aligned} \Vert {\textbf{P}}_N w \Vert _{L^p_T L^q}&\lesssim T^{\frac{1}{p}} N^{\alpha (s,p)} \big \{\Vert {\textbf{P}}_N w\Vert _{L^\infty _T H^s } + \Vert w_1\Vert _{L^\infty _T H^s } \Vert w_2\Vert _{L^\infty _T H^s } \\&\quad + \Vert w_3\Vert _{L^\infty _T H^s } \Vert w_4\Vert _{L^\infty _T H^s }+ \Vert {\textbf{P}}_N \phi \Vert _{L^{\infty }_T L^2 } \big \}. \end{aligned} \end{aligned}$$

The estimates in Lemma 2.6 give rise to the following bounds.

#### Lemma 2.7

Let $0<\delta <\infty$, $0<s\le \frac{1}{4}$, $N\in 2^{{\mathbb {N}}}$ such that $N\ge 2^6$, and $2\le q \le 4$ such that $(\frac{3}{2}-s)(\frac{1}{4}-\frac{1}{2q})-s <0$.

(i) If $v, v^\dagger$ are two solutions to the Cauchy problem of (2.5) with the same initial data $v_0\in H^s$, and we denote $\widetilde{v}= e^{-t {\mathcal {H}}\partial _x^2 }v(t)$ and ${\tilde{v}}^\dagger = e^{-t {\mathcal {H}}\partial _x^2 }v^\dagger (t)$, then

2.24 $$\begin{aligned} \Vert {\textbf{P}}_N \partial _t(\widetilde{v}-{\tilde{v}}^\dagger ) \Vert _{L^1_TL^2} \lesssim T N^{\frac{2}{q}+\frac{1}{2}} \big ( 1+\delta ^{-2}+\Vert v\Vert _{L^\infty _T H^s} + \Vert v^\dagger \Vert _{L^\infty _T H^s} \big )^3 \Vert v-v^\dagger \Vert _{L^\infty _T H^s}. \end{aligned}$$

(ii) If $w, w^\dagger$ are the gauge transformations of $v,v^\dagger$ in (i), respectively, and $\widetilde{w}= e^{ it\partial _x^2 }w(t)$ and $\widetilde{w}^{\dagger }= e^{it\partial _x^2 } w^\dagger (t)$, then

2.25 $$\begin{aligned} \begin{aligned} \Vert {\textbf{P}}_N \partial _t(\widetilde{w}-\widetilde{w}^{\dagger }) \Vert _{L^1_TL^2}&\lesssim T N^{\frac{2}{q}+\frac{1}{2}} \big ( 1+ \delta ^{-1}+T+\Vert v\Vert _{L^\infty _T H^s} + \Vert v^\dagger \Vert _{L^\infty _T H^s} \big )^6 \\&\quad \times \big ( \Vert w-w^\dagger \Vert _{L^\infty _T H^s} + \Vert v-v^\dagger \Vert _{L^\infty _T H^s} \big ). \end{aligned} \end{aligned}$$

#### Proof

The proofs of (i) and (ii) are almost exactly the same as those in [37, Lemma 2.11]. For (i), the only new estimate required is

$$\begin{aligned} \Vert e^{-t {\mathcal {H}}\partial _x^2} {\textbf{P}}_N {\mathcal {Q}}_{\delta } \partial _x(v-v^\dagger ) \Vert _{L^1_T L^2} \lesssim T \delta ^{-2} \Vert v-v^\dagger \Vert _{L^\infty _T L^2}, \end{aligned}$$

and for (ii), we only need to estimate

$$\begin{aligned} \Vert e^{ -it \partial _x^2 }{\textbf{P}}_N \partial _x{\textbf{P}}_{+,hi } ( e^{iF} {\mathcal {Q}}_{\delta } v -e^{iF^\dagger } {\mathcal {Q}}_{\delta } v^\dagger ) \Vert _{L^1_T L^2}. \end{aligned}$$

This is easily done by distributing the derivative, and using (2.3), Lemma 2.3, and (2.20). $\square$

The final result of this section is control of the solutions $v, v^{\dag }$ in terms of their gauge transformations $w,w^{\dag }$.

#### Lemma 2.8

Let $0<\delta <\infty$, $0 \le s <\frac{1}{2}$, $N\ge 10$ be a dyadic number, and $T>0$. If $v, v^\dagger$ are two solutions to (2.5) and $w,w^\dagger$ are the corresponding gauge transformations of $v,v^\dagger$ respectively, then

2.26 $$\begin{aligned}&\Vert {{\textbf {P}}}_{\le N} (v-v^\dagger ) \Vert _{C_T H^s} \nonumber \\  &\lesssim T \big \{ N^{\frac{3}{2}+s} (1+\Vert v\Vert _{C_TH^s} + \Vert v^\dagger \Vert _{C_T H^s} )^2 +\delta ^{-2}(1+\delta ^{-s})\big \} \Vert v-v^\dagger \Vert _{C_T L^2 } , \end{aligned}$$

2.27 $$\begin{aligned}&\begin{aligned}&\Vert {{\textbf {P}}}_{> N} (v-v^\dagger ) \Vert _{C_T H^s} \lesssim (1+\delta ^{-1}+\Vert v\Vert _{C_TH^s} + \Vert v^\dagger \Vert _{C_TH^s} )^5\\  &\quad \times \big ( \Vert w-w^\dagger \Vert _{C_TH^s} + \langle T \rangle (N^{s-\frac{1}{2}} + \Vert P_{>N} w^\dagger \Vert _{C_TH^s}) \Vert v-v^\dagger \Vert _{C_T L^2 } \big ). \end{aligned} \end{aligned}$$

#### Proof

We follow [37, Lemma 2.12]. For the low-frequencies in (2.26), we use the Duhamel formulation (2.21) for which the only new term can be estimated as follows using Lemma 2.3:

$$\begin{aligned} \Vert {\textbf{P}}_{\le N} {\mathcal {Q}}_{\delta }\partial _x(v-v^{\dagger } ) \Vert _{L^1_T H^s}\lesssim \delta ^{-2}(1+\delta ^{-s})T\Vert v- v^{\dagger } \Vert _{C_T L^2}. \end{aligned}$$

For the high-frequencies in (2.27), we use (2.20) whenever such a difference appears in the proof of [37, Lemma 2.12]. $\square$

## Normal form reductions on ${\mathbb {R}}$

The main goal of this section is to prove the following result which is an estimate controlling the difference of the gauged variables *w* in terms of the original variable *v*. This will provide a counterpart to Lemma 2.8.

### Proposition 3.1

Let $0<\delta <\infty$, $s_0<s\le \frac{1}{4},$ where $s_0$ is as in Theorem 1.1, $0<T<1$, and $M>1$. If $v, v^\dagger \in C_T H^s$ are two solutions to (2.5) with the same initial data and $w,w^\dagger$ are the corresponding gauge transformations of $v,v^\dagger$ respectively, then,

3.1 $$\begin{aligned} \begin{aligned} \Vert w-w^\dagger \Vert _{C_T H^s}&\lesssim (T M^\frac{3}{2} +M^{-\frac{1}{16} } ) (1+ \delta ^{-1} +\Vert v\Vert _{C_T H^s}+\Vert v^\dagger \Vert _{C_TH^s} )^{10} \\&\quad \quad \times (\Vert w-w^\dagger \Vert _{C_T H^s} +\Vert v-v^\dagger \Vert _{C_T H^s} ). \end{aligned} \end{aligned}$$

The proof follows the similar case for the BO equation dealt with in [37]. However, we must now control the additional terms arising from the depth parameter $0<\delta <\infty$. We postpone the proof of Proposition 3.1 as we must first make the necessary preparations for the normal form reductions we will employ. Given Proposition 3.1 and Lemma 2.8, we can give the proof of Theorem 1.2, which follows that in [37].

*Proof of Theorem* 1.2*when* ${\mathcal {M}}={\mathbb {R}}$. Fix $0<\delta <\infty$ and $s_0<s\le \frac{1}{4}$. It suffices to prove the unconditional well-posedness of solutions to the renormalised ILW equation (2.5). Given $u_0 \in H^{s}$, let $v\in C({\mathbb {R}},H^s)$ be the global solution to (2.5) constructed in [8, 15] such that $v\vert _{t=0}=u_0$. Suppose that there is an open interval *I* containing zero and another solution $v^{\dag }\in C(I;H^{s})$ to (2.5) such that $v^{\dag }\vert _{t=0}= u_0$. By time translation symmetry and time reversal symmetry of (2.5), it suffices to show $v \equiv v^\dagger \in C_T H^s$ for some small $T>0$. Let *w* and $w^\dagger$ be the gauge transforms of *v* and $v^\dagger$ given by (2.8), respectively. Fix $T_1\in I\cap (0,1)$ and let

$$\begin{aligned} K= (1+C_2) (1+\Vert v\Vert _{C_{T_1} H^s} + \Vert v^\dagger \Vert _{C_{T_1} H^s}+\delta ^{-1}), \end{aligned}$$

where $$C_2 $$ be the implicit constant in the estimate (2.27). Choose $$N\in 2^{{\mathbb {N}}}$$ so that

3.2 $$\begin{aligned} C_2 K^{5} (N^{s-\frac{1}{2}}+ \Vert {\textbf{P}}_{>\frac{N}{2}} w^\dagger \Vert _{C_{T_1} H^s}) \le \tfrac{1}{4}. \end{aligned}$$

Next, we choose $0<T<T_1$ such that

3.3 $$\begin{aligned} C_1 T \{ N^{\frac{3}{2}+s}K^2+ \delta ^{-2}\} \le \tfrac{1}{4}, \end{aligned}$$

where $C_1$ is the implicit constant in the estimate (2.26). Then, by (3.2), (3.3), and Lemma 2.8, we have

3.4 $$\begin{aligned} \Vert v-v^\dagger \Vert _{C_T H^s} \le 2C_{2}K^5 \Vert w-\widetilde{w}\Vert _{C_TH^s}. \end{aligned}$$

Now we use Proposition 3.1 to obtain the reverse difference estimate. Fix $0<\eta <\frac{1}{2}$ to be chosen later. We choose *M*, independently of *T*, so that $C_{3}M^{-\frac{1}{16}}K^{10} \le \eta$, where $C_3$ is the implicit constant in (3.1). By reducing *T*, if necessary, so that $C_{3}TM^{\frac{3}{2}}K^{10}\le \eta$, (3.1) implies

3.5 $$\begin{aligned} \Vert w-w^\dagger \Vert _{C_{T}H^{s}} \le \tfrac{2\eta }{1-2\eta }\Vert v-v^\dagger \Vert _{C_{T}H^{s}}. \end{aligned}$$

Finally, we choose $\eta$ sufficiently small so that

3.6 $$\begin{aligned} 2C_{2}K^{5}\tfrac{2\eta }{1-2\eta } \le \tfrac{1}{2}. \end{aligned}$$

Combining (3.4), (3.5), and (3.6) then shows that $v\equiv v^\dagger$ on [0, *T*], which completes the proof of Theorem 1.2 when ${\mathcal {M}}={\mathbb {R}}.$ $\square$

### Preparations for normal forms

Let $v\in C_{T}H^{s}$ be a solution to (2.5) and *w* be the gauge transformation of *v* defined in (2.8). Then, *w* solves (2.9). We pass to the interaction representation by defining $\widetilde{w}=e^{it \partial _x^2 }w$, $\widetilde{v}=e^{-t{\mathcal {H}}\partial _x^2}v(t)$, and $\widetilde{E}=e^{it \partial _x^2} E(t)$, where

3.7 $$\begin{aligned} E:=E_1(e^{iF},v)+E_2(e^{iF},v) \end{aligned}$$

and $E_1,E_2$ were defined in (2.10). Then we have

3.8 $$\begin{aligned} \widehat{\widetilde{w}}(t')\big \vert _{t'=0}^{t'=t}&= \hspace{-1mm}-2i \int _0^t \mathop {\mathrm {\int }}\limits _{\xi =\xi _{12}} e^{-it' \Omega (\xi ,\xi _1,\xi _2) } \tfrac{\xi \xi _2}{\xi _1} \sigma (\xi ,\xi _1,\xi _2) \widehat{\widetilde{w}} (t',\xi _1)\widehat{\widetilde{v}} (t',\xi _2) d\xi _1 dt' \nonumber \\&\quad + \int _{0}^{t} \widehat{\widetilde{E}}(t',\xi )dt', \end{aligned}$$

where $$\xi _{12}:=\xi _1+\xi _2$$,

3.9 $$\begin{aligned} \Omega (\xi ,\xi _1,\xi _2)= \xi |\xi |-\xi _1|\xi _1|-\xi _2|\xi _2| \,\, \text{ and } \,\, \sigma (\xi ,\xi _1,\xi _2)=\chi _+(\xi ) \widetilde{\chi }_+(\xi _1) {{\textbf {1}}}_{<0}(\xi _2). \end{aligned}$$

Here, $\chi _+$ is the symbol of ${\textbf{P}}_{+,hi }$ and $\widetilde{\chi }_+$ is a Schwartz function such that $\widetilde{\chi }_+=1$ on the support of $\chi _+$ and $\widetilde{\chi }_+=0$ on a neighbourhood of 0. Note that by the support restriction of $\sigma$, we have $\Omega (\xi ,\xi _1,\xi _2)=2\xi \xi _2$. On the other hand, we have

3.10 $$\begin{aligned} \begin{aligned} \widehat{\widetilde{v}}(t',\xi )\big \vert _{t'=0}^{t'=t}&=i\xi \int _0^t \int _{\xi =\xi _{12}} e^{-it'\Omega (\xi ,\xi _1,\xi _2)} \widehat{\widetilde{v}}(t',\xi _1) \widehat{\widetilde{v}}(t',\xi _2) d\xi _1 dt' \\&\quad + \int _0^t {\mathcal {F}}({\mathcal {Q}}_{\delta } \partial _xv)(t',\xi ) dt'. \end{aligned} \end{aligned}$$

We use the following lemma to justify the normal form steps for *v*.

#### Lemma 3.2

Let $v\in C_T L^2$ solve (2.5). Then, for each fixed $\xi \in {\mathbb {R}}$, the functions $t~\rightarrow ~\widehat{\widetilde{v}}(t,\xi ),\, \widehat{v}(t,\xi )$ are continuously differentiable.

#### Proof

It suffices to show that $\widehat{\widetilde{v}}(t,\xi )$ is continuously differentiable. From (3.10), we have

3.11 $$\begin{aligned} \partial _t\widehat{\widetilde{v}}(t,\xi ) = i\xi \int _{\xi =\xi _{12}} e^{-it\Omega (\xi ,\xi _1,\xi _2)} \widehat{\widetilde{v}}(t,\xi _1) \widehat{\widetilde{v}}(t,\xi _2) d\xi _1 + i \xi {\mathcal {Q}}_{\delta } (\xi ) \widehat{\widetilde{v}}(t,\xi ). \end{aligned}$$

The assumption $v\in C_{T}L^{2}_x$ with the dominated convergence theorem shows that

$$\begin{aligned} t \mapsto i\xi \int _{\xi =\xi _{12}} e^{-it\Omega (\xi ,\xi _1,\xi _2)} \widehat{\widetilde{v}}(t,\xi _1) \widehat{\widetilde{v}}(t,\xi _2) d\xi _1 \end{aligned}$$

is continuous for each fixed $\xi \in {\mathbb {R}}$. See [37, Lemma 3.2]. It follows from (3.11) that

$$\begin{aligned} \partial _t\big ( e^{-i \xi {\mathcal {Q}}_{\delta } (\xi ) t} \widehat{\widetilde{v}}(t,\xi ) \big )&= e^{-i \xi {\mathcal {Q}}_{\delta } (\xi ) t} \big [ \partial _t\widehat{\widetilde{v}}(t,\xi )-i \xi {\mathcal {Q}}_{\delta } (\xi ) \widehat{\widetilde{v}}(t,\xi ) \big ] \\&= e^{-i \xi {\mathcal {Q}}_{\delta } (\xi ) t} i \xi \int _{\xi =\xi _{12}} e^{-it\Omega }\, \widehat{\widetilde{v}}(t,\xi _1) \widehat{\widetilde{v}}(t,\xi _2) d\xi _1 . \end{aligned}$$

Here, the first equality is justified in the sense of temporal distributions for each fixed $\xi \in {\mathbb {R}}$, and the second equality shows that the map $t\mapsto e^{i \xi {\mathcal {Q}}_{\delta } (\xi ) t} \widehat{\widetilde{v}}(t,\xi )$ is $C^1$ for each fixed $\xi \in {\mathbb {R}}$. We thus conclude that $t\mapsto \widehat{\widetilde{v}}(t,\xi )$ is $C^{1}$ as well. $\square$

#### Remark 3.3

Given $v\in C_{T}L^{2}$ a solution to (2.5), let *w* be its gauge transformation according to (2.8). Contrary to the BO equation in [37], we are not able to show that $t\mapsto \widehat{w}(t,\xi )$ and $t\mapsto \widehat{\widetilde{w}}(t,\xi )$ are $C^{1}$ functions for each fixed $\xi \in {\mathbb {R}}$. The culprit is the term ${\mathcal {F}}E_2(e^{iF},v)(t,\xi )$ for each fixed $\xi \in {\mathbb {R}}$, as we only know $E_2(e^{iF},v)\in C_T L^2$ and not that $E_2(e^{iF},v)\in C_T L^1$. As we cannot apply the product rule directly in the normal form reductions for terms including $\widehat{\widetilde{w}}(t,\xi )$, we need extra ingredients in the normal form reductions below.

### First step of normal form reductions

Denote by ${\mathcal {N}}^{(1)}$ the bilinear operator

$$\begin{aligned} {\mathcal {F}}\{{\mathcal {N}}^{(1)}(\widetilde{w},\widetilde{v})\}(t,\xi )= -2i \int _{\xi =\xi _{12}} e^{-it \Omega (\xi ,\xi _1,\xi _2) } \frac{\xi \xi _2}{\xi _1} \sigma (\xi ,\xi _1,\xi _2) \widehat{\widetilde{w}} (t,\xi _1)\widehat{\widetilde{v}} (t,\xi _2) d\xi _1, \end{aligned}$$

and, for $M\ge 1$ to be chosen later, consider the decomposition ${\mathcal {N}}^{(1)}={\mathcal {N}}^{(1)}_{\le M} + {\mathcal {N}}^{(1)}_{> M}$, where ${\mathcal {F}}{\mathcal {N}}^{(1)}_{\le M}(\widetilde{w},\widetilde{v})(t,\xi )$ includes the additional restriction to the set $\{ \xi _1 \in {\mathbb {R}}: |\Omega (\xi ,\xi _1,\xi -\xi _1)|\le M\}$ and ${\mathcal {N}}_{>M}^{(1)}: = {\mathcal {N}}^{(1)}-{\mathcal {N}}^{(1)}_{\le M}$. We recall that due to the extra frequency restriction $\{|\Omega |\le M\}$, ${\mathcal {N}}^{(1)}_{\le M}$ can be controlled easily. See [37, Lemma 3.4] for a proof.

#### Lemma 3.4

If $s\ge 0$ and $0\le \theta <\frac{1}{2},$ then we have

$$\begin{aligned} \Vert {\mathcal {N}}^{(1)}_{\le M}(u_1,u_2) \Vert _{H^{s+\theta }} \lesssim M \Vert u_1\Vert _{H^s} \Vert u_2\Vert _{L^2}. \end{aligned}$$

There is no such control on ${\mathcal {N}}^{(1)}_{>M}$ and thus we proceed by an integration by parts in time which gains us a factor of $|\Omega |^{-1}$. For this purpose, we introduce the operator ${\mathcal {N}}^{(1)}_0$ by

$$\begin{aligned} {\mathcal {F}}\{ {\mathcal {N}}^{(1)}_0(u_1,u_2)\}(t,\xi ) =&\int _{\xi =\xi _{12}} e^{-it \Omega (\xi ,\xi _1,\xi _2) } \frac{1}{\xi _1} {{\textbf {1}}}_{\{|\Omega | > M\}}~\\  &\quad \sigma (\xi ,\xi _1,\xi _2) \widehat{u}_1 (t,\xi _1)\widehat{u}_2 (t,\xi _2) d\xi _1. \end{aligned}$$

Formally, integration by parts in time gives

3.12 $$\begin{aligned}&\int _0^t {\mathcal {F}}\{ {\mathcal {N}}_{>M}^{(1)}(\widetilde{w},\widetilde{v}) \} (t',\xi ) dt'\nonumber \\  &={\mathcal {F}}\{ {\mathcal {N}}^{(1)}_0(\widetilde{w},\widetilde{v}) \}(t',\xi ) \Big \vert ^{t'=t}_{t'=0}\nonumber \\  &\quad - \int _0^t \int _{\xi =\xi _{12}} \frac{\sigma e^{-it' \Omega }}{\xi _1} {{\textbf {1}}}_{\{|\Omega | > M\}} \partial _{t'} \big [ \widehat{\widetilde{w}}(t',\xi _1) \widehat{\widetilde{v}}(t',\xi _2) \big ]d\xi _1 dt' \nonumber \\  &= {\mathcal {F}}\{ {\mathcal {N}}^{(1)}_0(\widetilde{w},\widetilde{v}) \}(t',\xi ) \Big \vert ^{t'=t}_{t'=0} - \int _0^t {\mathcal {F}}\{ {\mathcal {N}}^{(1)}_0(\partial _{t'}\widetilde{w},\widetilde{v})\} (t',\xi )\nonumber \\  &\quad + {\mathcal {F}}\{ {\mathcal {N}}^{(1)}_0(\widetilde{w}, \partial _{t'}\widetilde{v})\}(t',\xi ) dt'. \end{aligned}$$

We can argue as in [37, subsection 3A] to justify the first equality in (3.12); namely, the switching of the *t* and $\xi _1$ integrals and the use of integration by parts in time. The new difficulty that arises in this paper is in justifying the second equality in (3.12). Namely, in view of Remark 3.3, we cannot apply the product rule to distribute the time derivative $\partial _{t'}$. To proceed we instead decompose $\widehat{\widetilde{w}}$ into a good and a bad term as in (3.16). For the contribution from the good term, we can apply the product rule and justify the series of equalities in (3.12). For the bad term, we instead directly prove the identity

$$\begin{aligned}&\int _0^t {\mathcal {F}}\{ {\mathcal {N}}_{>M}^{(1)}(B,\widetilde{v}) \} (t',\xi ) dt'\\&\quad = {\mathcal {F}}\{ {\mathcal {N}}^{(1)}_0(B,\widetilde{v}) \}(t',\xi ) \Big \vert ^{t'=t}_{t'=0} - \int _0^t {\mathcal {F}}\{ {\mathcal {N}}^{(1)}_0(\partial _{t'}B,\widetilde{v})\} (t',\xi )\\&\qquad + {\mathcal {F}}\{ {\mathcal {N}}^{(1)}_0(B, \partial _{t'}\widetilde{v})\}(t',\xi ) dt', \end{aligned}$$

where *B* is the bad part of *w*, thus bypassing the first equality in (3.12). This idea was inspired by [24, Appendix A]. We will also need to follow a similar justification procedure for the second round of integration by parts in Sect. 3.3.

We now continue with the first step of the normal form reductions. As in [37, subsection 3A], we first need to switch the order of integrations in $\xi _1$ and $t'$. We justify this use of Fubini’s theorem by using dyadic decompositions. We write $w_{N_1}:={\textbf{P}}_{N_1} w$ and $v_{N_2}:={\textbf{P}}_{N_2}v$, where $\widehat{w} = \sum _{N_1} \widehat{w}_{N_1}$ and $\widehat{v} = \sum _{N_2} \widehat{v}_{N_2}$ and consider

3.13 $$\begin{aligned} \begin{aligned}&\int _0^t {\mathcal {F}}\{ {\mathcal {N}}_{>M}^{(1)}\}(\widetilde{w},\widetilde{v}) (t',\xi ) dt' \\  &= -2i \xi \int _0^t \int _{\xi =\xi _{12}} e^{-it' \Omega } \frac{ \xi _2}{\xi _1} {{\textbf {1}}}_{\{|\Omega | > M\}}~ \sigma (\xi ,\xi _1,\xi _2) \sum _{N_1} \widehat{\widetilde{w}}_{N_1} (t',\xi _1) \sum _{N_2} \widehat{\widetilde{v}}_{N_2} (t',\xi _2) d\xi _1 dt'. \end{aligned} \end{aligned}$$

The following estimate allows us to justify the switching of the order of the integrals in $\xi _1$ and $t'$ and the summations over $N_1$ and $N_2$: for fixed $\xi \in {\mathbb {R}}$ and $0<t<T$, we have

3.14 $$\begin{aligned} \begin{aligned}&\sum _{N_1} \sum _{N_2} \int _0^t \int _{\xi =\xi _{12}} \Big | e^{-it' \Omega } \frac{\xi _2}{\xi _1} {\textbf{1}}_{\{|\Omega | > M\}}~ \sigma (\xi ,\xi _1,\xi _2) \widehat{\widetilde{w}} (t',\xi _1) \widehat{\widetilde{v}} (t',\xi _2) \Big | d\xi _1 dt' \\&\qquad \lesssim T \sum _{N_1} \sum _{N_2\lesssim N_1} \frac{N_2}{N_1}\Vert w_{N_1}\Vert _{L^\infty _T L^2} \Vert v_{N_2}\Vert _{L^\infty _T L^2}\\&\qquad \lesssim T \Vert w\Vert _{L^\infty _T H^s}\Vert v\Vert _{L^\infty _T L^2}, \end{aligned} \end{aligned}$$

for any $s>0$. Hence, by Fubini’s theorem, for fixed $\xi ,t$, we have

3.15 $$\begin{aligned} \begin{aligned} \text {LHS} (3.13)&= -2i \xi \sum _{N_1, N_2} \mathop {\mathrm {\int }}\limits _{\xi =\xi _{12}} \int _0^t e^{-it' \Omega } \frac{\xi _2}{\xi _1} {\textbf{1}}_{\{|\Omega | > M\}} \sigma \, \widehat{\widetilde{w}}_{N_1} (t',\xi _1) \widehat{\widetilde{v}}_{N_2} (t',\xi _2) dt' d\xi _1. \end{aligned} \end{aligned}$$

We decompose $\widetilde{w}$ as

3.16 $$\begin{aligned} \widetilde{w}= G+ B, \end{aligned}$$

where

3.17 $$\begin{aligned} \widehat{G}(t,\xi )&:= \hspace{-1mm} -2i \int _0^t\mathop {\mathrm {\int }}\limits _{\xi =\xi _{12}} e^{-it' \Omega } \tfrac{\xi \xi _2}{\xi _1} \sigma (\xi ,\xi _1,\xi _2) \widehat{\widetilde{w}} (t',\xi _1)\widehat{\widetilde{v}} (t',\xi _2) d\xi _1 dt' \nonumber \\&\quad + \int _0^t e^{- it' \xi ^2} \widehat{E}_1(e^{iF},v)(t',\xi ) dt', \end{aligned}$$

3.18 $$\begin{aligned} B(t,x)&:=\int _0^t e^{it' \partial _x^2} E_2(e^{iF}, v)(t',x)d t', \end{aligned}$$

and $E_1$ and $E_2$ were defined in (2.10). Then, arguing as in Lemma 3.2, the first term in the definition of $\widehat{G}(t,\xi )$ belongs to $C^{1}([0,T])$ for each fixed $\xi \in {\mathbb {R}}$. For the second term in $\widehat{G}(t,\xi )$, we note that (2.16) and (2.17) imply that $\widehat{E}_1(e^{iF},v)(t',\xi )\in C_{T}L^{\infty }_{\xi }$. Thus, $\widehat{G}(t,\xi ) \in C^{1}([0,T])$ for each fixed $\xi \in {\mathbb {R}}$. Hence, we can apply the integration by parts for the contribution with *G* and obtain that

3.19 $$\begin{aligned} \begin{aligned}&\int _0^t {\mathcal {F}}\{ {\mathcal {N}}_{>M}^{(1)}\}(G,\widetilde{v}) (t',\xi ) dt'\\  &= \sum _{N_1, N_2} \bigg \{ {\mathcal {F}}\{ {\mathcal {N}}^{(1)}_0(G_{N_1},\widetilde{v}_{N_2})\}(t',\xi ) \Big \vert ^{t'=t}_{t'=0} \\  &\quad + \int _{\xi =\xi _{12}} \int _0^t e^{-it' \Omega } \frac{1}{\xi _1} {{\textbf {1}}}_{\{|\Omega | > M\}}~ \sigma (\xi ,\xi _1,\xi _2) \partial _t\big (\widehat{G}_{N_1} (t',\xi _1) \widehat{\widetilde{v}}_{N_2} (t',\xi _2) \big ) dt' d\xi _1 \bigg \}, \end{aligned} \end{aligned}$$

where $G_{N}:={\textbf{P}}_{N} G.$ Here, using an estimate (for $t'=0,t$) analogous to (3.14), we see that the first term on the right-hand side of (3.19) is absolutely convergent. Moreover, by Lemma 3.2, we have $\widehat{\widetilde{v}}(t,\xi ), \widehat{G}(t,\xi )\in C^{1}_{T}$ for each fixed $\xi \in {\mathbb {R}}$, and thus

3.20 $$\begin{aligned} \begin{aligned} \text {LHS}(3.19)={\mathcal {F}}\{ {\mathcal {N}}^{(1)}_0(G,\widetilde{v})\}(t',\xi ) \Big \vert ^{t'=t}_{t'=0}&+ \sum _{N_1,N_2} \int _0^t {\mathcal {F}}\{ {\mathcal {N}}^{(1)}_0(\partial _{t'}G_{N_1},\widetilde{v}_{N_2})\}(t',\xi ) dt' \\&+ \sum _{N_1, N_2} \int _0^t {\mathcal {F}}\{{\mathcal {N}}^{(1)}_0(G_{N_1}, \partial _{t'}\widetilde{v}_{N_2})\}(t',\xi ) dt'. \end{aligned} \end{aligned}$$

For the bad contribution in (3.15), we need the following identity.

#### Lemma 3.5

Let $v\in C_{T}L^2$ be a solution to (3.10). For $N_1,N_2\in 2^{{\mathbb {N}}}$, define $\widetilde{v}_{N_2} = {\textbf{P}}_{N_2}e^{-t{\mathcal {H}}\partial _x^2}v(t)$ and $B_{N_1}:={\textbf{P}}_{N_1}B$, where $B\in C_{T} L^2$ is defined in (3.18). Then, for any $t_1,t_2\in [0,T]$, $M>0$, and fixed $\xi \in {\mathbb {R}}$,

3.21 $$\begin{aligned} \int _{t_1}^{t_2} \int _{\xi =\xi _{12}} e^{-it \Omega } f(t) d\xi _1 dt&= \bigg [ \int _{\xi =\xi _{12}} \frac{e^{-it \Omega } }{-i \Omega } f(t)d\xi _1 \bigg ] \bigg \vert ^{t=t_2}_{t=t_1}\nonumber \\&\quad -\int _{t_1}^{t_2} \int _{\xi =\xi _{12}} \frac{e^{-it \Omega } }{-i \Omega } g(t) d\xi _1 dt, \end{aligned}$$

where

$$\begin{aligned} f(t)&= -2i {\textbf{1}}_{\{\xi =\xi _{12}\}} \tfrac{\xi _2}{\xi _1} {\textbf{1}}_{\{|\Omega |> M\}}~ \sigma (\xi ,\xi _1,\xi _2) \widehat{B}_{N_1} (t,\xi _1) \widehat{\widetilde{v}}_{N_2} (t,\xi _2), \\&\begin{aligned} g(t)&= -2i {\textbf{1}}_{\{\xi =\xi _{12}\}} \tfrac{\xi _2}{\xi _1} {\textbf{1}}_{\{|\Omega | > M\}} \sigma (\xi ,\xi _1,\xi _2) \big [ \widehat{B}_{N_1} (t,\xi _1) \partial _t\widehat{\widetilde{v}}_{N_2} (t,\xi _2) \\&\quad +{\mathcal {F}}\{{\textbf{P}}_{N_1} e^{it\partial _x^2}E_2(e^{iF},v)\} (t,\xi _1) \widehat{\widetilde{v}}_{N_2} (t,\xi _2) \big ], \end{aligned} \end{aligned}$$

and $\Omega$ and $\sigma$ are defined in (3.9).

#### Proof

We adapt the approximation arguments from [24, Appendix A]. For $h\ne 0$ such that $t_1+h, t_2+h \in [0,T]$, we have the following identity

3.22 $$\begin{aligned} \begin{aligned}&\int _{t_1+h}^{t_2} \int _{\xi =\xi _{12}} \frac{e^{-it \Omega } -e^{-i(t-h) \Omega } }{ih \Omega } f(t) d\xi _1 dt\\&= \frac{1}{h} \int _{t_2}^{t_2+h} \int _{\xi =\xi _{12}} \frac{e^{-i(t-h) \Omega } }{i \Omega } f(t) d\xi _1 dt - \int _{t_1}^{t_1+h}\int _{\xi =\xi _{12}} \frac{e^{-it \Omega } }{i \Omega } f(t) d\xi _1 dt \\&\quad -\int _{t_1}^{t_2} \int _{\xi =\xi _{12}} \frac{ e^{-it \Omega }}{i \Omega } \frac{ f(t+h)-f(t) }{ h} d\xi _1 dt. \\ \end{aligned} \end{aligned}$$

To prove (3.21), we take limits in (3.22) as $h\rightarrow 0$ by proving the following:

3.23 $$\begin{aligned} \lim _{h\rightarrow 0}\int _{t_1+h}^{t_2} \int _{\xi =\xi _{12}} \frac{e^{-it \Omega } -e^{-i(t-h) \Omega } }{ih \Omega } f(t) d\xi _1 dt&= \int _{t_1}^{t_2} \int _{\xi =\xi _{12}} e^{-it \Omega } f(t) d\xi _1 dt, \end{aligned}$$

3.24 $$\begin{aligned} \lim _{h\rightarrow 0} \bigg [ \int _{t_0}^{t_0+h} \int _{\xi =\xi _{12}} \frac{e^{-i(t-h) \Omega } }{ih \Omega } f(t) d\xi _1 dt \bigg ] \bigg \vert ^{t_0=t_2}_{t_0=t_1}&= \bigg [ \int _{\xi =\xi _{12}} \frac{e^{-it \Omega } }{-i \Omega } f(t)d\xi _1 \bigg ] \bigg \vert ^{t=t_2}_{t=t_1}, \end{aligned}$$

3.25 $$\begin{aligned} \lim _{h\rightarrow 0} \int _{t_1}^{t_2} \int _{\xi =\xi _{12}} \frac{ e^{-it \Omega }}{i \Omega } \frac{ f(t+h)-f(t) }{ h} d\xi _1 dt&= \int _{t_1}^{t_2} \int _{\xi =\xi _{12}} \frac{e^{-it \Omega } }{-i \Omega } g(t) d\xi _1 dt. \end{aligned}$$

First, we verify (3.23). As $B_{N_1},v_{N_2}\in C_T L^2$ and $|\xi _1|\gtrsim |\xi _2|$ in the support of $\sigma$, we have $f \in C_T L^1_{\xi _1}.$

Thus, by the dominated convergence theorem, we have

$$\begin{aligned} \int _{\xi =\xi _{12} } \frac{ e^{-it \Omega } -e^{-i(t-h) \Omega } }{ih \Omega } f(t) d\xi _1 \longrightarrow \int _{\xi =\xi _{12}} e^{-it \Omega } f(t) d\xi _1, \text{ as } h\rightarrow 0 \end{aligned}$$

for each fixed $\xi \in {\mathbb {R}}$ and $t\in [t_1,t_2]$.

Applying the dominated convergence theorem again we obtain (3.23). For the limit (3.24), it suffices to show that for fixed $t_0\in [0,T],$

$$\begin{aligned}&\lim _{h\rightarrow 0} \int _{\xi =\xi _{12}} \Big ( \int _{t_0}^{t_0+h} \frac{e^{-i(t-h) \Omega } }{ih \Omega } f(t) dt - \frac{e^{-i t_0 \Omega } }{i \Omega } f(t_0) \Big ) d\xi _1 =0. \end{aligned}$$

Since $f\in C_T L^1_{\xi _1}$, we have

$$\begin{aligned} \bigg | \int _{\xi =\xi _{12}} \int _{t_0}^{t_0+h} \frac{e^{-i t_0 \Omega } }{i h\Omega } \big ( f(t) -f(t_0) \big ) dt d\xi _1 \bigg |&\lesssim \Vert f(t) -f(t_0)\Vert _{L^\infty (( t_0,t_0+h);L^1_{\xi _1})} \longrightarrow 0, \\ \bigg | \int _{\xi =\xi _{12}} \int _{t_0}^{t_0+h} \Big ( \frac{ e^{-i(t-h) \Omega } -e^{-i t_0 \Omega } }{ih \Omega } \Big ) f(t) d\xi _1 dt \bigg |&\lesssim h \Vert f\Vert _{C_TL^1_{\xi _1} } \longrightarrow 0, \end{aligned}$$

as $h \rightarrow 0$. Thus, the limit (3.24) holds true. Finally, we consider the limit (3.25). For $h\ne 0$, let $D_h$ denote the difference quotient operator, that is, $D_h f(t)=\tfrac{f(t+h)-f(t)}{h}.$ By using the facts $E_2(e^{if},v) \in C_T L^2$ and $\widetilde{v}\in C_T L^2,$ for fixed $\xi \in {\mathbb {R}}$ and any $t\in [0,T]$, as well as the identity $D_{h} [ f_1 f_2] = D_{h}[f_1]f_2 + f_1 D_h [f_2]$ for any two functions $f_1,f_2$, we have for sufficiently small *h*,

$$\begin{aligned} \begin{aligned} \bigg | \mathop {\mathrm {\int }}\limits _{\xi =\xi _{12}} \frac{ e^{-it \Omega }}{i \Omega } D_h f(t) d\xi _1 \bigg |&\lesssim \frac{1}{h}\int _t^{t+h} \Vert E_2(e^{iF},v)(t')\Vert _{L^2} \Vert v_{N_2}(t')\Vert _{L^2} \\&\quad + \Vert \partial _{t'}v_{N_2}(t')\Vert _{L^2} \Vert B(t')\Vert _{L^2}dt' \\&\lesssim \Vert v\Vert _{C_TL^2} \Vert E_2(e^{iF},v) \Vert _{C_TL^2}+ \Vert \partial _t\widetilde{v}_{N_2}\Vert _{C_T L^2} \Vert B\Vert _{C_TL^2} \\&\lesssim \Vert v\Vert _{C_TL^2} \Vert E_2(e^{iF},v) \Vert _{C_TL^2}+ N_2^{\frac{3}{2}} (1 +\delta ^{-1}\\&\quad + \Vert v \Vert _{L^\infty _T L^2})^2 \Vert B\Vert _{C_T L^2}, \end{aligned} \end{aligned}$$

where we used (3.10), and the Bernstein inequality in the third inequality.

Then, by the dominated convergence theorem, it suffices to show that for each fixed $\xi \in {\mathbb {R}}$, we have

3.26 $$\begin{aligned}&\lim _{h\rightarrow 0} \int _{\xi =\xi _{12}} -2i \tfrac{ e^{-it \Omega }}{i \Omega } \tfrac{\xi _2}{\xi _1} {\textbf{1}}_{\{|\Omega | > M\}}~ \sigma \big \{ D_h \widehat{B}_{N_1} (t,\xi _1) \nonumber \\&\quad - e^{-it\xi _1^2}\widehat{E}_2(e^{iF},v)(t,\xi _1) \big \} \widehat{\widetilde{v}}_{N_2} (t,\xi _2) d\xi _{1} =0, \end{aligned}$$

3.27 $$\begin{aligned}&\lim _{h\rightarrow 0} \int _{\xi =\xi _{12}} -2i \tfrac{ e^{-it \Omega }}{i \Omega } \tfrac{ \xi _2}{\xi _1} {\textbf{1}}_{\{|\Omega | > M\}}~ \sigma \widehat{B}_{N_1} (t,\xi _1) \big \{ D_h \widehat{\widetilde{v}}_{N_2}(t,\xi _2) \nonumber \\&\quad - \partial _t\widehat{\widetilde{v}}_{N_2}(t,\xi _2) \big \} d\xi _{1} = 0. \end{aligned}$$

By using that $E_2(e^{iF},v) \in C_T L^2$ and $\widetilde{v}\in C_T L^2,$ for fixed $\xi \in {\mathbb {R}}$ and $t\in [0,T]$, we have

$$\begin{aligned} \bigg | \int _{\xi =\xi _{12}}&-2i \frac{ e^{-it \Omega }}{i \Omega } \frac{ \xi _2}{\xi _1}{\textbf{1}}_{\{|\Omega | > M\}}~ \sigma (\xi ,\xi _1,\xi _2) \big \{ D_h \widehat{B}_{N_1} (t,\xi _1) - \widehat{E}_2(e^{iF},v)(t,\xi _1) \big \} \widehat{\widetilde{v}}_{N_2} (t,\xi _2) d\xi _{1} \bigg | \\&\lesssim \Vert \widetilde{v}\Vert _{C_T L^2} \frac{1}{h} \int _{t}^{t+h} \Vert E_2(e^{iF},v)(t',x)-e^{-it \xi _1^2}E_2(e^{iF},v)(t,x)\Vert _{L^2} dt' \longrightarrow 0, \end{aligned}$$

as $h \rightarrow 0$, which proves (3.26). For (3.27), by Lemma 3.2, the equation (3.10), $\widetilde{v}\in C_TL^2$, and the Bernstein inequality, it holds that

$$\begin{aligned} \Vert D_h \widehat{\widetilde{v}}_{N_2}(t,\xi ) - \partial _t\widehat{\widetilde{v}}_{N_2}(t,\xi ) \Vert _{L^2_{\xi }}&\le \frac{1}{h} \bigg \Vert \int _t^{t+h} \big ( \partial _{t'}\widehat{\widetilde{v}}_{N_2}(t',\xi ) - \partial _t\widehat{\widetilde{v}}_{N_2}(t,\xi ) \big ) dt' \bigg \Vert _{L^{2}_{\xi }} \\&\lesssim (\delta ^{-2}+ N_2^{\frac{3}{2}}\Vert v\Vert _{C_TL^2}) \frac{1}{h} \int _t^{t+h} \Vert v(t)-v(t')\Vert _{L^2} dt' \longrightarrow 0, \end{aligned}$$

as $h\rightarrow 0.$ Thus, since $E_2 \in C_T L^2$, we get (3.27). This completes the proof of (3.21). $\square$

Thus, by Lemma 3.5, we obtain

3.28 $$\begin{aligned} \begin{aligned}&\int _0^t {\mathcal {F}}\{ {\mathcal {N}}_{>M}^{(1)}\}(B,\widetilde{v}) (t',\xi ) dt'\\  &= \sum _{N_1, N_2} {\mathcal {F}}\{ {\mathcal {N}}^{(1)}_0(B_{N_1},\widetilde{v}_{N_2})\}(t',\xi ) \Big \vert ^{t'=t}_{t'=0} \\  &\quad + \sum _{N_1, N_2} \int _{\xi =\xi _{12}} \int _0^t \frac{\sigma e^{-it' \Omega } }{\xi _1} {{\textbf {1}}}_{\{|\Omega |> M\}} {\mathcal {F}}\{ {{\textbf {P}}}_{N_1} e^{it' \partial _x^2}E_2(e^{iF}, v)\}(t',\xi _1) \widehat{\widetilde{v}}_{N_2} (t',\xi _2) d\xi _1 dt' \\  &\quad + \sum _{N_1, N_2} \int _{\xi =\xi _{12}} \int _0^t \frac{\sigma e^{-it' \Omega } }{\xi _1} {{\textbf {1}}}_{\{|\Omega | > M\}}\widehat{B}_{N_1} (t',\xi _1) \partial _{t'}\widehat{\widetilde{v}}_{N_2} (t',\xi _2) d\xi _1 dt' . \end{aligned} \end{aligned}$$

Finally, combining (3.20), (3.28), and using (3.16), we now formally have the identity

3.29 $$\begin{aligned} \begin{aligned}&\int _0^t {\mathcal {F}}\{{\mathcal {N}}_{>M}^{(1)}\}(\widetilde{w},\widetilde{v}) (t',\xi ) dt'\\  &={\mathcal {F}}\{ {\mathcal {N}}^{(1)}_0(\widetilde{w},\widetilde{v})\}(t',\xi ) \Big \vert ^{t'=t}_{t'=0} + \sum _{N_1, N_2} \int _0^t {\mathcal {F}}\{ {\mathcal {N}}^{(1)}_0( \partial _{t'}\widetilde{w}_{N_1} ,\widetilde{v}_{N_2})\}(t',\xi ) dt' \\  &\quad + \sum _{N_1,N_2} \int _0^t {\mathcal {F}}( {\mathcal {N}}^{(1)}_0 ( \widetilde{w}_{N_1} , \partial _{t'}\widetilde{v}_{N_2}))(t',\xi ) dt'. \end{aligned} \end{aligned}$$

The boundary term in (3.29) admits a good estimate. See [37, Lemma 3.6].

#### Lemma 3.6

Let $s\ge 0$ and $0\le \theta <\frac{1}{2}$. Then, for each fixed time $t\in [0,T]$,

$$\begin{aligned} \Vert {\mathcal {N}}^{(1)}_0(u_1,u_2) \Vert _{H^{s+\theta } } \lesssim M^{-\frac{1}{8}+\frac{\theta }{4}} \Vert u_1\Vert _{H^s} \Vert u_2\Vert _{L^2}. \end{aligned}$$

As pointed out in [37, Remark 3.5], the estimates in Lemma 2.7 are not good enough to ensure that the last two terms on RHS (3.29) are absolutely summable in $N_1$. However, we continue to proceed with the normal form reductions for each fixed $N_1$ and $N_2$. Eventually, we will be able to perform the summation in $N_1$ and thus make fully rigorous the justification of the normal form equation we obtain. See the proof of Proposition 3.1.

We now substitute the equations (3.8), and (3.10) for $\partial _t\widetilde{w}$, and $\partial _t\widetilde{v}$, respectively, into the last two terms of (3.29). First, we consider the substitution of $\partial _{t'}\widetilde{w}$ in (3.8), which is justified by Lemma 3.6 and Lemma 2.7. We will rewrite the frequency $N_1$ of $\widetilde{w}$ as $N_{12}$ and the frequency $N_2$ of $\widetilde{v}$ as $N_3$. For fixed $N_{12},N_3$ and $\xi \in {\mathbb {R}}$, the term ${\mathcal {F}}\{{\mathcal {N}}^{(1)}_0(\partial _{t'}\widetilde{w}_{N_{12} }, \widetilde{v}_{N_3} ) \}(t',\xi ) dt'$ in (3.29) is equal to

3.30 $$\begin{aligned}&\int _{\xi =\eta +\xi _3} \int _0^t e^{-it' \Omega (\xi ,\eta ,\xi _3)} \frac{{{\textbf {1}}}_{\{|\Omega |>M\}}}{\eta } \sigma (\xi ,\eta ,\xi _3) \partial _{t'}\widehat{\widetilde{w}}_{N_{12}}(t',\eta ) \widehat{\widetilde{v}}_{N_3}(t',\xi _3) dt' d\eta \nonumber \\  &\begin{aligned}&= \int _{\xi =\eta +\xi _3} \int _0^t e^{-it' \Omega (\xi ,\eta ,\xi _3)} \frac{{{\textbf {1}}}_{\{|\Omega |>M\}}}{\eta } \sigma (\xi ,\eta ,\xi _3) \psi _{N_{12}}(\eta ) \\  &\times \bigg ( \int _{\eta =\xi _{12} } e^{-it'\Omega (\eta ,\xi _1,\xi _2)} \frac{2\eta \xi _2}{i\xi _1} \sigma (\eta ,\xi _1,\xi _2) \widehat{\widetilde{w}}(\xi _1) \widehat{\widetilde{v}}(\xi _2) d\xi _1 +\widehat{E}(\eta ) \bigg ) \widehat{\widetilde{v}}_{N_3}(t',\xi _3) dt' d\eta \end{aligned} \end{aligned}$$

3.31 $$\begin{aligned}&\quad \begin{aligned}&= \sum _{N_1, N_2} \int _{\xi =\xi _{123}} \int _0^t e^{-it'\Omega ^{(2)}_{1}(\xi ,\xi _1,\xi _2,\xi _3) } m^{(2)}_{1}(\xi ,\xi _1,\xi _2,\xi _3) \widehat{\widetilde{w}}_{N_1}(\xi _1) \widehat{\widetilde{v}}_{N_2}(\xi _2) \widehat{\widetilde{v}}_{N_3}(\xi _3) dt' d\xi _1d\xi _2\\  &\quad + \int _{\xi =\eta +\xi _3} \int _0^t e^{-it' \Omega (\xi ,\eta ,\xi _3)} \frac{{{\textbf {1}}}_{\{|\Omega |>M\}}}{\eta } \sigma (\xi ,\eta ,\xi _3) \psi _{N_{12}} (\eta ) \widehat{E}(\eta ) \widehat{\widetilde{v}}_{N_3}(t',\xi _3)dt' d\eta , \end{aligned} \end{aligned}$$

where *E* was defined in (3.7), $\xi _{123}:=\xi _1+\xi _2+\xi _3$, and

3.32 $$\begin{aligned} \begin{aligned} \Omega ^{(2)}_1(\xi ,\xi _1,\xi _2,\xi _3)&:=\Omega (\xi ,\xi _{12},\xi _3) +\Omega (\xi _{12},\xi _2,\xi _3)=2\xi \xi _3+2\xi _{12}\xi _2, \\ m^{(2)}_1 (\xi ,\xi _1,\xi _2,\xi _3)&:=-2i\frac{\xi _2}{\xi _1} {\textbf{1}}_{\{|\Omega |>M\}} \sigma (\xi ,\xi _{12},\xi _3) \sigma (\xi _{12},\xi _1,\xi _2) \psi _{N_{12} }(\xi _{12}) \prod _{k=1}^3 \psi _{N_k} (\xi _k). \end{aligned} \end{aligned}$$

Here, we discuss the rigour of the last equality (3.31). Since, for fixed $t\in [0,T)$ and $\xi \in {\mathbb {R}},$ by Cauchy-Schwarz and Lemma 2.4,

$$\begin{aligned}&\int _{\xi =\eta +\xi _3} \int _0^t \Big | \tfrac{{\textbf{1}}_{\{|\Omega |>M\}}}{\eta } \sigma (\xi ,\eta ,\xi _3) \psi _{N_{12}} (\eta ) \widehat{E}(\eta ) \widehat{\widetilde{v}}(t',\xi _3) \Big |dt' d\eta \\&\qquad \lesssim \tfrac{T}{N_{12}}\Vert E\Vert _{C_T L^2} \Vert v\Vert _{C_T L^2} \lesssim \tfrac{T}{N_{12}}(1+\delta ^{-1}+ \Vert v\Vert _{C_T L^2})^3 \Vert v\Vert _{C_T L^2}, \end{aligned}$$

so we can decompose

3.33 $$\begin{aligned}&\begin{aligned} (3.30)&= \int _{\xi =\eta +\xi _3} \int _0^t e^{-it'\Omega (\xi ,\eta ,\xi _3)} \frac{{\textbf{1}}_{ \{|\Omega |>M\}}}{\eta } \sigma (\xi ,\eta ,\xi _3) \psi _{N_{12}}(\eta ) \\&\quad \times \int _{\eta =\xi _{12} } e^{-it'\Omega (\eta ,\xi _1,\xi _2)} \frac{2\eta \xi _2}{i\xi _1} \sigma (\eta ,\xi _1,\xi _2) \widehat{\widetilde{w}}(\xi _1) \widehat{\widetilde{v}}(\xi _2) d\xi _1 \widehat{\widetilde{v}}_{N_3}(t',\xi _3) dt' d\eta \end{aligned} \nonumber \\&\qquad \qquad + \int _{\xi =\eta +\xi _3} \int _0^t e^{-it' \Omega (\xi ,\eta ,\xi _3)} \frac{{\textbf{1}}_{\{|\Omega |>M\}}}{\eta } \sigma (\xi ,\eta ,\xi _3) \psi _{N_{12}} (\eta ) \widehat{E}(\eta ) \widehat{\widetilde{v}}_{N_3}(t',\xi _3)dt' d\eta . \end{aligned}$$

Next, to rewrite (3.33) as (3.31), dyadically decompose (3.33) into dyadic intervals of $\xi _1$ and $\xi _2$, and pull the summations outside of the integrals using Fubini’s theorem. This hinges on the fact that the sums and integrals in (3.31) converge absolutely; see [37, (3-30), p.306].

For fixed dyadics $N_{12},N_1,N_2,N_3$, we define the multilinear operator ${\mathcal {N}}^{(2)}_{1}$ by

$$\begin{aligned}&\quad {\mathcal {F}}\{{\mathcal {N}}^{(2)}_{1}(\widetilde{w},\widetilde{v},\widetilde{v}) \}(t,\xi ) = \int _{\xi =\xi _{123}} e^{-it\Omega ^{(2)}_{1} } m^{(2)}_{1}(\xi ,\xi _1,\xi _2,\xi _3) \widehat{\widetilde{w}}(\xi _1) \widehat{\widetilde{v}}(\xi _2) \widehat{\widetilde{v}}(\xi _3) d\xi _1d\xi _2. \end{aligned}$$

Now, we consider the substitution of (3.10) for $\partial _t\widetilde{v}$ in (3.29). In this case, we rewrite $N_2$ as $N_{23}$. For fixed $N_1$ and $N_{23}$, we have

3.34 $$\begin{aligned} \begin{aligned}&\quad {\mathcal {N}}^{(1)}_0 (\widetilde{w}_{N_1},\partial _t\widetilde{v}_{N_{23} } )= {\mathcal {N}}^{(1)}_0 (\widetilde{w}_{N_1}, {\textbf{P}}_{N_{23}} e^{-t{\mathcal {H}}\partial _x^2 } \partial _x(v^2) ) + {\mathcal {N}}^{(1)}_0 (\widetilde{w}_{N_1}, {\textbf{P}}_{N_{23}} {\mathcal {Q}}_{\delta } \partial _x\widetilde{v}). \end{aligned} \end{aligned}$$

Here, the first term on the right-hand side of (3.34) satisfies

3.35 $$\begin{aligned} \begin{aligned}&{\mathcal {F}}\{{\mathcal {N}}^{(1)}_0 (\widetilde{w}_{N_1},{{\textbf {P}}}_{N_{23}} e^{-t{\mathcal {H}}\partial _x^2 } \partial _x(v^2)) \}(t,\xi ) \\  &= -i \sum _{N_2} \sum _{N_3} \int _{\xi =\xi _{123}} e^{-i \Omega ^{(2)}_2(\xi ,\xi _1,\xi _2,\xi _3) } m^{(2)}_*(\xi ,\xi _1,\xi _2,\xi _3) \widehat{\widetilde{w}}_{N_1}(\xi _1) \widehat{\widetilde{v}}_{N_2}(\xi _2) \widehat{\widetilde{v}}_{N_3}(\xi _3) d\xi _1 d\xi _2, \end{aligned} \end{aligned}$$

where

3.36 $$\begin{aligned} \Omega ^{(2)}_2&=\Omega (\xi ,\xi _1,\xi _{23})+\Omega (\xi _{23},\xi _2,\xi _3), \nonumber \\ m^{(2)}_*(\xi ,\xi _1,\xi _2,\xi _3)&=\tfrac{\xi _{23}}{\xi _1} {\textbf{1}}_{\{|\Omega (\xi ,\xi _1,\xi _{23}) |>M\}} \sigma (\xi ,\xi _1,\xi _{23}) \psi _{N_{23}}(\xi _{23}) \psi _{N_1} (\xi _1)\psi _{N_2} (\xi _2)\psi _{N_3} (\xi _3). \end{aligned}$$

The justification of (3.35) follows similar to the justification of (3.31) and we thus omit it. See [37, (3.36)]. Following [37], we decompose $\{(\xi ,\xi _1,\xi _2,\xi _3) \in {\mathbb {R}}^4\}=R^{(2)}_{\le M}\cup R_{>M}^{(2)},$ where

$$\begin{aligned} R^{(2)}_{\le M}&= \{|\xi _{12}|\le 1\} \cup \{ |\Omega ^{(2)}_2(\xi ,\xi _1,\xi _2,\xi _3)|\le M \} \quad \text {and} \quad R_{>M}^{(2)} := {\mathbb {R}}^{4} \backslash R_{\le M}^{(2)}. \end{aligned}$$

We define

3.37 $$\begin{aligned} m_{\le M}^{(2)} = m^{(2)}_*{\textbf{1}}_{R^{(2)}_{\le M}} \quad \text {and}\quad m_{2}^{(2)}= m^{(2)}_* {\textbf{1}}_{R^{(2)}_{>M}} . \end{aligned}$$

For fixed $N_2,N_3$, we define ${\mathcal {N}}_{\le M}^{(2)}$ and ${\mathcal {N}}_{2}^{(2)}$ by

$$\begin{aligned}&F {\mathcal {N}}_{\le M}^{(2)}(v_1,v_2,v_3)(t,\xi ) = -i \int _{\xi =\xi _{123}} e^{-i \Omega ^{(2)}_2(\xi ,\xi _1,\xi _2,\xi _3) }\\&\quad m^{(2)}_{\le M}(\xi ,\xi _1,\xi _2,\xi _3) \widehat{\widetilde{w}}(\xi _1) \widehat{\widetilde{v}}(\xi _2) \widehat{\widetilde{v}}(\xi _3) d\xi _1 d\xi _2,\\&F {\mathcal {N}}_{2}^{(2)}(v_1,v_2,v_3)(t,\xi ) = -i \int _{\xi =\xi _{123}} e^{-i \Omega ^{(2)}_2(\xi ,\xi _1,\xi _2,\xi _3) } \\&\quad m^{(2)}_{2}(\xi ,\xi _1,\xi _2,\xi _3) \widehat{\widetilde{w}}(\xi _1) \widehat{\widetilde{v}}(\xi _2) \widehat{\widetilde{v}}(\xi _3) d\xi _1 d\xi _2. \end{aligned}$$

Therefore, we may write

$$\begin{aligned} {\mathcal {N}}^{(1)}_0 (\widetilde{w}_{N_1}, {\textbf{P}}_{N_{23}} e^{-t{\mathcal {H}}\partial _x^2 } \partial _x(v^2) ) = \sum _{N_2} \sum _{N_3} \big \{ {\mathcal {N}}^{(2)}_{\le M}(\widetilde{w},\widetilde{v},\widetilde{v}) + {\mathcal {N}}^{(2)}_{2}(\widetilde{w},\widetilde{v},\widetilde{v}) \big \} . \end{aligned}$$

Note that for simplicity we have omitted the subscripts $N_{j}$ on the right-hand side for clarity. For the contribution due to ${\mathcal {N}}^{(2)}_{\le M}$, we recall the following lemma, see [37, Lemma 3.7].

#### Lemma 3.7

If $s\ge 0$ and $0\le \theta <\min (s,\frac{1}{2})$, then we have

$$\begin{aligned} \Vert {\mathcal {N}}^{(2)}_{\le M} (v_1, v_2 ,v_3) \Vert _{H^{s+\theta } } \lesssim M^\frac{3}{2} \Vert v_1\Vert _{H^s} \Vert v_2\Vert _{H^s} \Vert v_3\Vert _{H^s}. \end{aligned}$$

In summary, after the first step of the normal form reductions we have arrived at

3.38 $$\begin{aligned}&\widetilde{w}(t')\big \vert _{t'=0}^{t'=t} = \int _0^t {\mathcal {N}}_{\le M}^{(1)}(\widetilde{w},\widetilde{v})(t') dt' + \int _0^t \widetilde{E}(t') dt' + {\mathcal {N}}^{(1)}_{0} (\widetilde{w},\widetilde{v})(t') \Big |^{t'=t}_{t'=0} \nonumber \\&-\sum _{N_{12}} \sum _{N_3\lesssim N_{12}} \bigg \{ \sum _{\begin{array}{c} N_1, N_2 \\ N_2\lesssim N_1 \end{array} } \int ^t_0 {\mathcal {N}}^{(2)}_1 (\widetilde{w},\widetilde{v},\widetilde{v})(t')dt' + \int _0^t {\mathcal {N}}^{(1)}_0({\textbf{P}}_{N_{12}} \widetilde{E}, \widetilde{v}_{N_3}) dt' \bigg \} \nonumber \\&-\sum _{N_1} \sum _{N_{23}\lesssim N_1} \bigg \{ \sum _{N_2, N_3} \int ^t_0 {\mathcal {N}}^{(2)}_{\le M} (\widetilde{w},\widetilde{v},\widetilde{v}) +{\mathcal {N}}^{(2)}_{2} (\widetilde{w},\widetilde{v},\widetilde{v}) dt' +\int _0^t {\mathcal {N}}^{(1)}_0(\widetilde{w}_{N_1},{\textbf{P}}_{N_{23}} {\mathcal {Q}}_{\delta } \partial _x\widetilde{v}) dt' \bigg \}. \end{aligned}$$

Relative to [37, (3.42)], the only new terms here are the second and fifth (implicitly through (3.7)), and the last term. We show that the summations in the fifth and the last terms converge absolutely. By Lemma 3.6, for $t\in [0,T]$, $s> 0$ and $0<\theta _1<\frac{1}{2}$ and $\theta _2>0$ such that $\theta _2<\min (s, \tfrac{1}{2} -\theta _1)$, we have

3.39 $$\begin{aligned} \begin{aligned}&\sum _{N_{12}} \sum _{N_3\lesssim N_{12} } \int _0^t \Vert {\mathcal {N}}^{(1)}_{0} ({{\textbf {P}}}_{N_{12}} {\tilde{E}}, \widetilde{v}_{N_3} ) (t') \Vert _{H^{s+\theta _1} } dt'\\  &\lesssim T M^{-\frac{1}{8}+\frac{\theta _1+\theta _2}{4}} \sum _{N_{12}} \sum _{N_3\lesssim N_{12} } \langle N_{12} \rangle ^{-\theta _2} \Vert {{\textbf {P}}}_{N_{12}} \widetilde{E}\Vert _{L^\infty _T H^s} \Vert v\Vert _{ L^\infty _T L^2}\\  &\lesssim T M^{-\frac{1}{8}+\frac{\theta _1+\theta _2}{4}} \Vert \widetilde{E}\Vert _{L^\infty _T H^s} \Vert v\Vert _{L^\infty _T L^2} \\  &\lesssim T M^{-\frac{1}{8}+\frac{\theta _1+\theta _2}{4}} \Vert v\Vert _{L^{\infty }_{T}L^2}^{2} \big \{ 1+\Vert v\Vert _{L^{\infty }_{T}L^2}+\delta ^{-1}(1+\delta ^{-1-s})(1+\Vert v\Vert _{L^{\infty }_{T}H^{s}})^2\big \}, \end{aligned} \end{aligned}$$

where in the last inequality we used Lemma 2.4 and (2.18). Similarly, by Lemma 3.6 and Lemma 2.3, for $t\in [0,T]$, $s> 0$, we have

3.40 $$\begin{aligned} \sum _{\begin{array}{c} N_1, N_{23} \\ N_{23}\lesssim N_1 \end{array}} \int _0^t \Vert {\mathcal {N}}^{(1)}_0 (\widetilde{w}_{N_1},{\textbf{P}}_{N_{23}}{\mathcal {Q}}_{\delta } \partial _x\widetilde{v})\Vert _{H^{s+\theta _1}} dt' \lesssim T M^{-\frac{1}{8}+\frac{\theta _1+\theta _2}{4}} \delta ^{-2} \Vert w\Vert _{L^\infty _T H^s} \Vert v\Vert _{L^\infty _T L^2}. \end{aligned}$$

It remains to justify that the summations over the terms ${\mathcal {N}}^{(1)}_{1}$ and ${\mathcal {N}}^{(2)}_2$ are finite and this involves a second round of integration by parts which is the role of the next section.

### Second step of the normal form reductions

First, we note that due to the frequency restrictions, we have $m_j^{(2)}\in L^{\infty }({\mathbb {R}}^4)$ and $|\Omega _{j}^{(2)}|>M$ for each $j=1,2$. This is clear for $j=1$ from (3.32) and can be deduced for $j=2$ by further splitting $R^{(2)}_{>M}$ into sub-regions depending on the signs of $\xi _2$ and $\xi _3$.

We then apply integration by parts to estimate the terms ${\mathcal {N}}^{(2)}_{j}$, $j=1,2$. To justify this, we argue as we did in the previous section for the first step. Namely, we need to decompose $\widetilde{w}$ according to (3.16). As the good term *G* is continuously differentiable in *t*, we can apply integration by parts and the product rule. For the bad term $B\in C_T L^2$, we use the following lemma, which is analogous to Lemma 3.5.

#### Lemma 3.8

Let $v\in C_{T}L^2$ be a solution to (3.10). For $N_1,N_2,N_3\in 2^{{\mathbb {N}}}$, define $\widetilde{v}_{N_{\ell }} = {\textbf{P}}_{N_{\ell }}e^{-t{\mathcal {H}}\partial _x^2}v(t)$ for $\ell =2,3$, and $B_{N_1}={\textbf{P}}_{N_1}B$, where $B\in C_{T} L^2$ is defined in (3.18). Then, for any $t_1,t_2\in [0,T]$, $M>0$, fixed $\xi \in {\mathbb {R}}$, and for each $j=1,2$, it holds that

3.41 $$\begin{aligned} \begin{aligned}&\int _{t_1}^{t_2} \int _{\xi =\xi _{123}} e^{-it \Omega ^{(2)}_j } f_j(t) d\xi _1d\xi _2 dt \\  &= \bigg [ \int _{\xi =\xi _{12}} \frac{e^{-it \Omega ^{(2)}_j} }{-i \Omega ^{(2)}_j } f_j(t)d\xi _1 \bigg ] \bigg \vert ^{t=t_2}_{t=t_1} -\int _{t_1}^{t_2} \int _{\xi =\xi _{12}} \frac{e^{-it \Omega ^{(2)}_j} }{-i \Omega ^{(2)}_j } g_j(t) d\xi _1d\xi _2 dt, \end{aligned} \end{aligned}$$

where

$$\begin{aligned} f_j(t)&= m_j^{(2)} (\xi ,\xi _1,\xi _2,\xi _3) \widehat{B}_{N_1} (t,\xi _1) \widehat{\widetilde{v}}_{N_2} (t,\xi _2) \widehat{\widetilde{v}}_{N_3} (t,\xi _3), \\ g_j(t)&= m_j^{(2)} (\xi ,\xi _1,\xi _2,\xi _3) \widehat{B}_{N_1} (t,\xi _1) \partial _t\widehat{\widetilde{v}}_{N_2} (t,\xi _2) \widehat{\widetilde{v}}_{N_3} (t,\xi _3)\\&\quad + m_j^{(2)} (\xi ,\xi _1,\xi _2,\xi _3) \widehat{B}_{N_1} (t,\xi _1) \widehat{\widetilde{v}}_{N_2} (t,\xi _2) \partial _t\widehat{\widetilde{v}}_{N_3} (t,\xi _3)\\&\quad +m_j^{(2)} (\xi ,\xi _1,\xi _2,\xi _3) {\mathcal {F}}\{{\textbf{P}}_{N_1}E_2(e^{iF},v)\} (t,\xi _1) \widehat{\widetilde{v}}_{N_2} (t,\xi _2) \widehat{\widetilde{v}}_{N_3} (t,\xi _3), \end{aligned}$$

$\Omega _{1}^{(2)}$ and $m_{1}^{(2)}$ are defined in (3.32), $\Omega _{1}^{(2)}$ is defined in (3.36), and $m_{2}^{(2)}$ is defined in (3.37).

#### Proof

We argue as in the proof of Lemma 3.8, taking limits in the analog of (3.22). These limits exist and yield (3.41) since we have $\widehat{\widetilde{v}}_N,\partial _t\widehat{\widetilde{v}}_N\in C_TL^2_\xi \cap C_T L^1_\xi$ and $E_2(e^{iF},v)\in C_T L^2$ and we use the dominated convergence theorem.

For the reader’s convenience, we show a limit analogous to (3.23): for fixed $\xi$,

3.42 $$\begin{aligned} \int _{t_1+h}^{t_2} \int _{\xi =\xi _{123}} \frac{e^{-it \Omega ^{(2)}_j} -e^{-i(t-h) \Omega ^{(2)}_j} }{ih \Omega ^{(2)}_j } f_j(t) d\xi _1 d\xi _2 dt \rightarrow \int _{t_1}^{t_2} \int _{\xi =\xi _{12}} e^{-it \Omega ^{(2)}_j } f_j(t) d\xi _1 d\xi _2 dt, \end{aligned}$$

as $$h\rightarrow 0$$.

For each $j=1,2,3$, Hölder’s inequality implies

$$\begin{aligned} \int _{\xi =\xi _{123} } |f_j(t)| d\xi _1d\xi _2&\lesssim \big ( |\widehat{B}_{N_1} | *|\widehat{\widetilde{v}}_{N_2} | *|\widehat{\widetilde{v}}_{N_3} |\big )(\xi ) \lesssim \Vert B_{N_1}\Vert _{L^2 } \Vert \widetilde{v}_{N_2}\Vert _{L^2 } \Vert \widehat{\widetilde{v}}_{N_3}\Vert _{L^1 }\\&\lesssim N_3 T \Vert E_2(e^{iF},v)\Vert _{C_T L^2 } \Vert \widetilde{v}_{N_2}\Vert _{C_T L^2 } \Vert \widehat{\widetilde{v}}_{N_2}\Vert _{C_T L^2}, \end{aligned}$$

uniformly for $t\in [0,T]$, and thus $f_j\in C_T L^1_{\xi _1,\xi _2}$ which is enough to prove (3.42). It is straightforward then to adapt this idea to prove analogs of the limits (3.24) and (3.25). We omit the details. $\square$

Applying integration by parts for the contributions with *G* and Lemma 3.8 for those with *B*, we obtain

3.43 $$\begin{aligned} \begin{aligned} \int _0^t {\mathcal {N}}_j^{(2)}(\widetilde{w},\widetilde{v},\widetilde{v}) dt'&= \big [ {\mathcal {N}}^{(2)}_{j,0}(\widetilde{w},\widetilde{v},\widetilde{v}) \big ] \big |^{t'=t}_{t'=0} - \int _0^t {\mathcal {N}}^{(2)}_{j,0}( \partial _t\widetilde{w},\widetilde{v},\widetilde{v}) dt'\\&\quad - \int _0^t {\mathcal {N}}^{(2)}_{j,0}( \widetilde{w}, \partial _t\widetilde{v},\widetilde{v}) dt' -\int _0^t {\mathcal {N}}^{(2)}_{j,0}( \widetilde{w},\widetilde{v}, \partial _t\widetilde{v}) dt' , \end{aligned} \end{aligned}$$

where for $j=1,2$, we defined

$$\begin{aligned} \begin{aligned} {\mathcal {F}}\{{\mathcal {N}}_{j,0}^{(2)}(v_1,v_2,v_3) \}(t,\xi )&=\int _{\xi =\xi _{123}} e^{-it\Omega ^{(2)} _j(\xi ,\xi _1,\xi _2,\xi _3) }\nonumber \\&\quad \frac{m_j^{(2)} (\xi ,\xi _1,\xi _2,\xi _3)}{-i \Omega ^{(2)}_j (\xi ,\xi _1,\xi _2,\xi _3)} \prod _{k=1}^3 \widehat{v}_{k} (\xi _k) d\xi _1 d\xi _2. \end{aligned} \end{aligned}$$

Provided that $s>s_0$, we can successfully control all the terms in (3.43) and gain a small power of the largest dyadic frequency to justify the convergence of the sums in (3.38).

#### Lemma 3.9

Let $0<\delta <\infty$ and $s_0<s\le \frac{1}{4}$, where $s_0$ is as in Theorem 1.1. Assume $0<T<1$ and let $v, v^{\dag }\in C_T H^s$ be two solutions to (2.5) with the same initial data. Then, there exists $\theta >0$ such that for any $\varepsilon >0$ sufficiently small

$$\begin{aligned}&\big \Vert {\mathcal {N}}^{(2)}_j(\widetilde{w},\widetilde{v},\widetilde{v}) - {\mathcal {N}}^{(2)}_j(\widetilde{w}^{\dagger },{\tilde{v}}^\dagger ,{\tilde{v}}^\dagger ) \big \Vert _{L^1_T H^{s+\theta } } \\&\quad \lesssim N_{\max }^{-\varepsilon } (M^{-\frac{1}{8}} +T ) ( 1 + \delta ^{-1}+ \Vert v\Vert _{L^\infty _T H^s} +\Vert v^\dagger \Vert _{L^\infty _T H^s} )^8 \\&\quad \quad \times ( \Vert w-w^\dagger \Vert _{L^\infty _T H^s} + \Vert v-v^\dagger \Vert _{L^\infty _T H^s}), \end{aligned}$$

for each $j=1,2$, where ${\tilde{v}}^\dagger = e^{-t{\mathcal {H}}\partial _x^2} \widetilde{v}$ and $\widetilde{w}^{\dagger }= e^{ it\partial _x^2 } w^\dagger$ and $N_{\max }:=\max _{j=1,2,3} N_j$.

#### Proof

We estimate the right-hand side of (3.43). The boundary term is handled by

$$\begin{aligned} \max _{j=1,2}\Vert {\mathcal {N}}^{(2)}_{j,0}(v_1,v_2,v_3) \Vert _{H^{s+\frac{1}{2}}} \lesssim M^{-\frac{1}{4}+\varepsilon } \Vert v_1\Vert _{H^s} \Vert v_2\Vert _{L^2} \Vert v_3\Vert _{L^2} \end{aligned}$$

for any $s\ge 0$ and $\varepsilon >0$. See [37, Lemma 3.9] for a proof. The remaining terms in RHS(3.43) are then estimated as in the proof of [37, Corollary 3.11] where the differences $\partial _t(\widetilde{v}- {\tilde{v}}^\dagger )$ and $\partial _t(\widetilde{w}-\widetilde{w}^{\dagger })$ are now controlled by (2.24) and (2.25), respectively. Consequently, we have the same restriction $s>s_0$ as in [37, Corollary 3.11]. Note that in [37] the case $j=2$ requires further decomposition but since these terms are unaltered in our setting, we have omitted such detail. $\square$

#### Proof of Proposition 3.1

By the above normal form reductions, we arrive at the normal form equation for the gauged variables $\widetilde{w}$ given in (3.38) with (3.43). As each of the summations converge absolutely, when we consider the difference $\widetilde{w}$ and $\widetilde{w}^{\dag }$, we can use telescoping sums to rewrite each of the differences. Then, the estimate (3.1) follows from Lemmas 2.4, 2.5, 3.4, 3.6, 3.7, (3.39), (3.40) and Lemma 3.9.

## On the periodic setting

In this section, we consider the ILW on the circle and detail the necessary modifications needed to complete the proof of Theorem 1.2 in the setting ${\mathcal {M}}={\mathbb {T}}$. Namely, establishing unconditional uniqueness for $s_0<s <1/2$, where $s_0$ is as in Theorem 1.1.

We first discuss the gauge transformation in the periodic case which is slightly different to that on the line in Sect. 2.2. Fix $0<\delta <\infty$. Let $u\in C([0,T];L^2({\mathbb {T}}))$ be a solution (1.1) for some $T>0$ and let *v* be its corresponding Galilean transform as in (2.4). As *v* is a distributional solution to (2.5) it follows that its mean $\int _{\mathbb {T}}v(t,x)dx$ is conserved. Upon considering the transformation $v(t,x) \mapsto v(t,x-\tfrac{t}{\pi } \int _{{\mathbb {T}}} u_0 ) - \tfrac{1}{2\pi } \int _{{\mathbb {T}}} u_0(x)dx$ we may assume that *v* is of mean-zero for all times *t*. We define the primitive *F* of *v* via its Fourier coefficients:

$$\begin{aligned} \widehat{F} (t,0) = 0, \quad \text {and} \quad \widehat{F}(t,n) = \tfrac{1}{in} \widehat{v}(t,n) \quad \text {for} \,\, n\in {\mathbb {Z}}\setminus \{0\}. \end{aligned}$$

As *v* is mean-zero, we have $F=\partial _x^{-1}v$. Note that *F* satisfies a version of Lemma 2.5 trivially due to Bernstein’s inequality. Moreover, it solves the equation

$$\begin{aligned} \partial _tF -{\mathcal {H}}\partial _x^2 F =v^2-{\textbf{P}}_{0}(v^2) +{\mathcal {Q}}_{\delta } v, \end{aligned}$$

where ${\textbf{P}}_0 f:= \widehat{f}(0)$. In the periodic case, $e^{iF}\in L^2({\mathbb {T}})$ so ${\textbf{P}}_{\pm } (e^{iF})$ are well-defined. We define the gauge transformed variable

4.1 $$\begin{aligned} w=\partial _x{\textbf{P}}_{+} e^{iF} \end{aligned}$$

and we see that *w* satisfies

4.2 $$\begin{aligned} \begin{aligned} \partial _tw- {\mathcal {H}}\partial _x^2 w&=-2 \partial _x{\textbf{P}}_{+}(\partial _x^{-1} w \cdot {\textbf{P}}_{-} \partial _xv )+ i\partial _x{\textbf{P}}_{+} (e^{iF} {\mathcal {Q}}_{\delta } v)-i{\textbf{P}}_{0}(v^2)w . \end{aligned} \end{aligned}$$

Similar to the BO case in [37], we cannot assume that the $L^2$-norm of *v* is conserved in time. Thus, it is beneficial to remove the last term on the right-hand side of (4.2) by using a further gauge transformation

$$\begin{aligned} z(t,x) :=w(t,x) e^{i \gamma (t)} \quad \text {where} \quad \gamma (t)=\gamma (t)[v] : = \int _{0}^{t} {\textbf{P}}_{0}(v^2(t'))dt'. \end{aligned}$$

Then, *z* solves

$$\begin{aligned} \partial _tz- {\mathcal {H}}\partial _x^2 z&= -2 \partial _x{\textbf{P}}_{+,hi } (\partial _x^{-1} z \cdot {\textbf{P}}_{-} \partial _xv ) +E_{3}[z,v] + e^{i\gamma }E_{4}[e^{iF}, v], \end{aligned}$$

where

$$\begin{aligned} E_{3}[f,g]: = -2{\textbf{P}}_{+}{\textbf{P}}_{lo } ( \partial _x^{-1} f \cdot {\textbf{P}}_- \partial _xg) \quad \text {and} \quad E_{4}[f,g] = i\partial _x{\textbf{P}}_{+}(f {\mathcal {Q}}_{\delta } g). \end{aligned}$$

Note that $E_3$ and $e^{i\gamma } E_4$ satisfy similar estimates to (2.11) and (2.12), respectively. In particular, for each fixed $n\in {\mathbb {Z}}$, Cauchy-Schwarz and Lemma 2.3 give

$$\begin{aligned} |{\mathcal {F}}\{ e^{i\gamma [f]}\partial _x{\textbf{P}}_{+}[ e^{iF[g]}{\mathcal {Q}}_{\delta } h]\}(n)| \lesssim |n| \Vert e^{iF[g]}\Vert _{L^{2}} \Vert {\mathcal {Q}}_{\delta } h\Vert _{L^2} \lesssim \delta ^{-1}\Vert h\Vert _{L^2}, \end{aligned}$$

which implies that the maps $t\mapsto \widehat{z}(t,n), \widehat{\widetilde{z}}(t,n)$, where $\widetilde{z}:=e^{-t{\mathcal {H}}\partial _x^2}z$, are continuously differentiable for each fixed *n*. This means that we can apply the product rule in our normal form reductions and thus we do not need to consider a decomposition of the form (3.16).

The refined Strichartz estimates and hence Lemma 2.7 are slightly different in the periodic case. We give them here to also indicate to indicate the location of the $\delta$-dependence in the estimate which comes from an application of Lemma 2.3. Apart from this, the argument is exactly the same as that in [37, Lemma 5.1] so we omit the proof. We point out that it is likely possible to adapt the strategy of Kishimoto [24] to (2.5) and establish unconditional uniqueness for $\frac{1}{6}<s<\frac{1}{2}$ on ${\mathbb {T}}$ without having to use the refined Strichartz estimates.

### Lemma 4.1

Let $0\le s\le \frac{1}{4}$, $0<\delta <\infty$, $N\in 2^{{\mathbb {N}}}$, $T>0$, and $2\le p\le 4$. If *u* is a solution to $\partial _tu-{\mathcal {H}}\partial _x^2 u= \partial _x(u_1 u_2) +{\mathcal {Q}}_{\delta } \partial _xu_3.$ Then, we have

4.3 $$\begin{aligned} \Vert {\textbf{P}}_N u\Vert _{L^{p}_{T,x}} \lesssim T^{\frac{1}{p}}N^{\beta (s,p)}\big ( \Vert {\textbf{P}}_{N}u\Vert _{L^{\infty }_{T}H^{s}} +\Vert u_1\Vert _{L^{\infty }_{T}H^{s}}\Vert u_2\Vert _{L^{\infty }_{T}H^{s}} + \delta ^{-2}\Vert u_3\Vert _{L^{\infty }_{T}L^2} \big ), \end{aligned}$$

where $$\beta (s,p) = \big ( \tfrac{3}{2}-s\big ) \big (\tfrac{1}{4}-\tfrac{1}{2p}\big )-s.$$

Using (4.3), we can derive analogous results to (2.23), (2.24), and (2.25). All of the normal form considerations are then carried out exactly as in Sect. 3 and the proofs of Theorem 1.2 and Corollary 1.3 then follow the same arguments. This completes the proof of the unconditional uniqueness for ILW in $C_{T} H^{s}({\mathbb {T}})$ for all $s_0<s<\frac{1}{4}$. Finally, we note that unconditional uniqueness in the range $\frac{1}{4} \le s <\frac{1}{2}$ then follows from the fact that unconditional uniqueness in $C_{t}H^{s}$ implies unconditional uniqueness in $C_{t}H^{s'}$ for any $s' > s$.

## Footnotes

1 Here, we endow $C({\mathbb {R}};H^{s}({\mathcal {M}}))$ with the compact-open topology in time.
